# Glutathione-dependent redox balance characterizes the distinct metabolic properties of follicular and marginal zone B cells

**DOI:** 10.1038/s41467-022-29426-x

**Published:** 2022-04-04

**Authors:** Davide G. Franchina, Henry Kurniawan, Melanie Grusdat, Carole Binsfeld, Luana Guerra, Lynn Bonetti, Leticia Soriano-Baguet, Anouk Ewen, Takumi Kobayashi, Sophie Farinelle, Anna Rita Minafra, Niels Vandamme, Anaïs Carpentier, Felix K. Borgmann, Christian Jäger, Ying Chen, Markus Kleinewietfeld, Vasilis Vasiliou, Michel Mittelbronn, Karsten Hiller, Philipp A. Lang, Dirk Brenner

**Affiliations:** 1 Experimental and Molecular Immunology, Department of Infection and Immunity, Luxembourg Institute of Health, Esch-sur-Alzette, Luxembourg; 2 Immunology & Genetics, Luxembourg Centre for Systems Biomedicine, University of Luxembourg, 7, Avenue des Hauts Fourneaux, Esch-sur-Alzette, Luxembourg; 3 Department of Molecular Medicine II, Medical Faculty, Heinrich-Heine-University, 40225 Düsseldorf, Germany; 4 National Data Mining and Modelling for Biomedicine, VIB Center for Inflammation Research, Ghent, Belgium; 5 Department of Applied Mathematics, Computer Science and Statistics, Ghent University, Ghent, Belgium; 6 National Center of Pathology (NCP), Laboratoire National de Santé (LNS), Dudelange, Luxembourg; 7 Luxembourg Center of Neuropathology (LCNP), Dudelange, L-3555 Luxembourg; 8 Luxembourg Centre for Systems Biomedicine, University of Luxembourg, 7 Avenue des Hauts Fourneaux, Esch-sur-Alzette, Luxembourg; 9 Department of Environmental Health Sciences, Yale School of Public Health, New Haven, CT USA; 10 VIB Laboratory of Translational Immunomodulation, VIB Center for Inflammation Research (IRC) Hasselt University, Diepenbeek, Belgium; 11 Department of Immunology, Biomedical Research Institute, Hasselt University, Diepenbeek, Belgium; 12 Faculty of Science, Technology and Medicine, University of Luxembourg, Esch-sur-Alzette, Luxembourg; 13 Department of Life Sciences and Medicine (DLSM), University of Luxembourg, Esch-sur-Alzette, Luxembourg; 14 Luxembourg Centre for Systems Biomedicine (LCSB), University of Luxembourg, Esch-sur-Alzette, L-4362 Luxembourg; 15 Department of Oncology (DONC), Luxembourg Institute of Health (LIH), Luxembourg, L-1526 Dudelange, Luxembourg; 16 Department for Bioinformatics and Biochemistry, Braunschweig Integrated Center of Systems Biology (BRICS), Technische Universität Braunschweig, Rebenring 56, 38106 Braunschweig, Germany; 17 Odense Research Center for Anaphylaxis (ORCA), Department of Dermatology and Allergy Center, Odense University Hospital, University of Southern Denmark, Odense, Denmark

**Keywords:** Follicular B cells, Humoral immunity, Marginal zone B cells, Antimicrobial responses

## Abstract

The metabolic principles underlying the differences between follicular and marginal zone B cells (FoB and MZB, respectively) are not well understood. Here we show, by studying mice with B cell-specific ablation of the catalytic subunit of glutamate cysteine ligase (*Gclc*), that glutathione synthesis affects homeostasis and differentiation of MZB to a larger extent than FoB, while glutathione-dependent redox control contributes to the metabolic dependencies of FoB. Specifically, *Gclc* ablation in FoB induces metabolic features of wild-type MZB such as increased ATP levels, glucose metabolism, mTOR activation, and protein synthesis. Furthermore, *Gclc*-deficient FoB have a block in the mitochondrial electron transport chain (ETC) due to diminished complex I and II activity and thereby accumulate the tricarboxylic acid cycle metabolite succinate. Finally, *Gclc* deficiency hampers FoB activation and antibody responses in vitro and in vivo, and induces susceptibility to viral infections. Our results thus suggest that *Gclc* is required to ensure the development of MZB, the mitochondrial ETC integrity in FoB, and the efficacy of antiviral humoral immunity.

## Abstract (web summary)

Follicular and marginal zone B (FoB and MZB, respectively) cells have divergent metabolic characteristics. Here the authors show that deficiency of glutamate cysteine ligase (Gclc), the enzyme for glutathione synthesis, differentially impacts FoB and MZB homeostasis, while specifically impeding FoB activation and downstream antiviral immunity.

## Introduction

B cells regulate many functions required for immune homeostasis and can present antigens very efficiently through their major histocompatibility complexes to T cells^1,2^. Furthermore, B cells can release immunomodulatory cytokines that are critical for the normal immune system maintenance, and differentiate into effector subsets that secrete polarized cytokines depending on the environment^3^. However, the principal function of a B lymphocyte is to secrete antibodies that provide humoral immunity. Antibody protection is a key component of the innate and adaptive phases of the immune response and is mediated mainly by two distinct splenic B cell subsets: marginal zone B cells (MZB) and follicular B cells (FoB)^4–6^. Previous studies have described developmental, phenotypic, functional and transcriptomic differences between MZB and FoB^7–14^. Moreover, distinct homeostatic control mechanisms regulate FoB and MZB distribution in the spleen^15,16^. FoB persist in the follicles in a quiescent state as they recirculate until activated by recognition of the antigen by their B cell receptors and the T cell-mediated cognate help, whereupon they proliferate and undergo germinal center (GC) reactions^6,17^. In contrast, MZB do not recirculate between lymphoid organs, are proximal to blood vessels, and can self-propagate^18^. Furthermore, MZB possess innate-like properties and are activated earlier during an immune challenge than FoB^8^. Nevertheless, like FoB, MZB can undergo GC reactions^19,20^.

Redox balance is essential for maintaining cellular signaling and activation^21^. Glutathione (GSH) is a key intracellular antioxidant that scavenges excess reactive oxygen species (ROS)^22–24^, and it is an important molecule for the regulation of lymphocytes activation^25–27^. Moreover, early studies on HIV-1-infected subjects associated GSH deficiency with a poor lymphocytic response and impaired survival following HIV-1 infections^28–30^, implying a role for GSH in disease. In B cells, ROS are instrumental in regulating activation^31,32^, but the contribution to B cell subsets and functions of mitochondrial ROS (mtROS), which are generated principally within mitochondria, is poorly understood. It is, therefore, possible that redox thresholds in B cells are subset-specific, and that these thresholds mediate homeostatic functions that could account for the differing properties of MZB and FoB. To this end, Muri et al. have previously shown that GSH-dependent glutathione peroxidase 4 (Gpx4) activity is critical for MZB compared to FoB^33^. However, the precise role of the tripeptide GSH in B cell metabolism remains unknown.

The mechanistic target of rapamycin (mTOR) signaling is a key modulator of anabolic and catabolic reactions^34^, which in turn could affect ROS balance. mTOR has emerged as a crucial control point for B cell functions^35^. Previous reports have shown that relatively high levels of mTOR complex 1 (mTORC1) signaling prevail in MZB^36,37^, but that the maturation of FoB downregulates the mTORC1/Akt pathway^38^. However, other groups have shown that efficient mTORC1 suppression is necessary to avoid MZB loss^39^. Because mTORC1 is a well-known regulator of metabolic functions, these findings imply that specific metabolic programs may underlie the unique characteristics of different B cell subsets.

Here, we report that blocking GSH synthesis by ablation of *Gclc* causes loss of MZB. In the absence of GSH synthesis, FoB upregulate mTORC1 and reprogram their metabolism towards glycolysis, which is similar to the metabolic program of wild-type MZB. However, GSH-deficient FoB accumulate defective mitochondria and do not activate upon viral challenge. In summary, our analysis shows that GSH is crucial for the development of MZB and for the control of mitochondrial metabolic functions in FoB. Therefore, our results demonstrate a subset-specific role for GSH in controlling the redox balance underlying the metabolic properties between MZB and FoB.

## Results

### FoB and MZB exhibit distinct glutathione-based redox dependencies

**Fig. 1:**
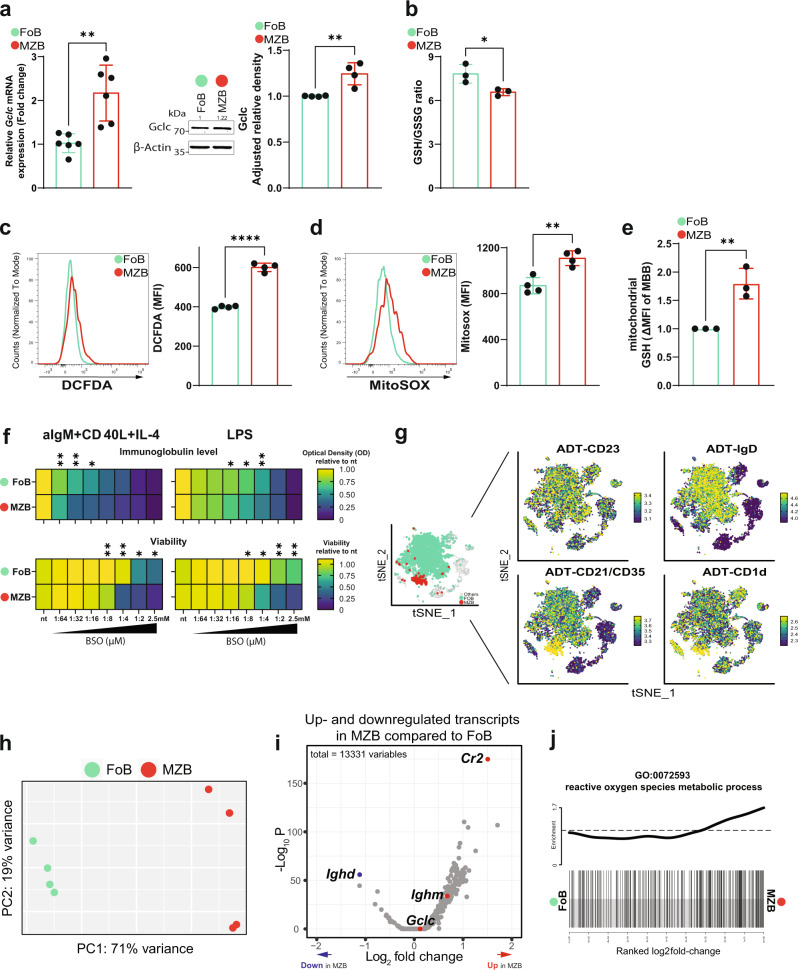
GSH-dependent redox activity differs between FoB and MZB. **a** Left: RT-qPCR of *Gclc* mRNA expression in resting *Gclc*-sufficient FoB (green) and MZB (red) isolated from spleen of B6 mice (*n* = 5 animals examined over three independent experiments). Middle: representative blot of Gclc protein from total cell lysis of resting B6 FoB and MZB (*n* = 3 animals examined over three independent experiments). Right: relative density of Gclc protein expression in resting B6 FoB and MZB (*n* = 3 animals examined over three independent experiments). **b** Luminescence-based quantitation of intracellular GSH/GSSG ratio in resting FoB and MZB isolated from B6 mice (*n* = 3 animals examined over three independent experiments). (**c**–**d**) Representative histogram and quantitation of DCFDA (**c**) and MitoSOX (**d**) staining for intracellular ROS and mitochondrial (mt) ROS detection in splenic B6 FoB and MZB (gated as in Supplementary Fig. 1a). **e** Flow-cytometry-based quantitation of monobromobimane (MBB) for the detection of GSH in purified Tom20^+^ mitochondria from B6 FoB and MZB (gated as in Supplementary Fig. 1a) (*n* = 3–4 animals examined over two independent experiments). **f** Heatmap showing relative expression of immunoglobulin level (top) and viability (bottom) of 4d activated B6 FoB and MZB with increasing concentration of BSO. **g** tSNE plot showing SCINA assignments of *Gclc*-sufficient FoB and MZB (left) isolated from spleen of *Gclc*^*fl/fl*^ mice (gated as in Supplementary Fig. 1b) and based on the ADT signals of CD23, IgD, CD21/35, and CD1d markers (right). Green: FoB; Red: MZB; Grey: unassigned B cells. Data are pooled from 4 *Gclc*^*fl/fl*^ mice. Sidebars represent the ADT expression scale. **h** PCA plot showing distinct transcriptome patterns of *Gclc*-sufficient FoB and MZB transcriptomes from 4 *Gclc*^*fl/fl*^ mice. **i** Volcano plot of differentially expressed genes in resting *Gclc*-sufficient FoB vs. MZB. Data represent up- and downregulated transcripts in *Gclc*-sufficient MZB compared to *Gclc*-sufficient FoB. Red/blue dots indicate upregulated/downregulated signature genes in resting *Gclc*-sufficient MZB compared to FoB. **j** Barcode plot showing enrichment of GO:0072593 gene list (reactive oxygen species metabolic processes) in resting *Gclc*-sufficient MZB compared to FoB. For all applicable figure panels, data are mean ± SD and each dot represents one single mouse. Significance (*P*) was calculated with unpaired t-test, except for (**f**) (2 way ANOVA). *P ≤ 0.05; **P ≤ 0.01; ****P ≤ 0.0001.

To dissect the redox state of MZB and FoB in relation to the main antioxidant GSH, we studied the expression of *Gclc*, which is necessary for GSH synthesis^24^, in isolated MZB and FoB from spleens of C57BL/6J (B6 or wild-type) mice. We found that both mRNA and protein levels of Gclc were significantly higher in B6 MZB compared to B6 FoB (Fig. 1a). However, the ratio of reduced to oxidized GSH (GSH/GSSG) was lower in B6 MZB than in B6 FoB indicating increased ROS in MZB (Fig. 1b). Indeed, staining of CD23^high^ CD21/35^low^ FoB and CD23^low^ CD21/35^high^ MZB (gated as shown in Supplementary Fig. 1a) with DCFDA, a fluorescent sensor of hydrogen peroxide^40^, revealed increased ROS in B6 MZB compared to B6 FoB (Fig. 1c). Metabolic reactions intrinsically generate oxygen radicals, and mitochondria are the major source of metabolic ROS^41^. Evaluation of mtROS with MitoSOX staining^42^ indicated that B6 MZB generate more mtROS compared to FoB (Fig. 1d). Accordingly, the absolute levels of GSH were increased in isolated mitochondria (gated as shown in Supplementary Fig. 1a) from B6 MZB compared to B6 FoB (Fig. 1e). These data suggest that MZB experience higher ROS generation and GSH consumption, a notion that correlates with a more sustained metabolic activity of MZB at steady-state.

Given the potential role of GSH in the regulation of immune functions^43,44^ and the distinct expression of *Gclc* in mature B cell subsets, we studied FoB and MZB activation in the presence of buthionine sulfoximine (BSO), a Gclc inhibitor^45^. In order to assess activation, B6 FoB and MZB where stimulated with anti-IgM, CD40 ligand and IL-4 or LPS for 4 days and treated with BSO. We found that wild-type MZB were more susceptible to Gclc inhibition as shown by a stronger BSO concentration-dependent decrease in viability and total immunoglobulin secretion in the culture supernatants when compared to FoB (Fig. 1f). Altogether, these data indicate that GSH is differentially important in FoB and MZB at steady state, and that MZB show higher dependency on GSH upon in vitro activation.

To explore the relevance of GSH to the distinct redox properties of FoB and MZB in vivo, we generated B cell-specific *Gclc*-deficient mice by crossing *Gclc*^*fl/fl*^ mice with *Mb1-Cre*^*+*^ mice (*Gclc*^*fl/fl*^ *Mb1-Cre*^*+*^), in which the *Cre* recombinase gene is expressed under the control of the B cell-specific promoter *Mb1*^46^. First, we aimed to compare the transcriptome of MZB and FoB. Therefore, we FACS-sorted splenic B cells from *Gclc*^*fl/fl*^ mice (gated as in Supplementary Fig. 1b) and applied single-cell CITE-sequencing (CITE-seq) proteomics^47^. FoB and MZB express specific surface markers, such as CD19^+^ CD23^high^ CD21/35^low^ IgD^high^ and CD19^+^ CD23^low^ CD21/35^high^ CD1d^high^, respectively^7,48–50^. By using CD23, IgD, CD21/CD35, and CD1d antibody-derived tag (ADT) signals, we identified FoB and MZB in the CITE-seq dataset, assigned each cell type using SCINA (Fig. 1g)^51^ and conducted downstream analyses. The gene expression PCA plot confirmed transcriptomic-wide differences between GSH-sufficient *Gclc*^*fl/fl*^ MZB and *Gclc*^*fl/fl*^ FoB (Fig. 1h). Differential expression analysis indicated a general upregulation of transcription in *Gclc*^*fl/fl*^ MZB compared to *Gclc*^*fl/fl*^ FoB (Fig. 1i), consistent with previous reports^10,37^. Moreover, together with MZB-related signature transcripts such as *Cr2* and *Ighm*, *Gclc* was also upregulated in *Gclc*^*fl/fl*^ MZB compared to FoB (Fig. 1i), validating the dataset. Additionally, gene ontology analysis identified that *Gclc*-sufficient MZB transcriptomes showed upregulation of genes that are related to ROS metabolic processes when compared to *Gclc*-sufficient FoB (Fig. 1j and Supplementary Data 1). These data indicate that genes associated with metabolic ROS generation tend to be upregulated in MZB compared to FoB, suggesting that differences in the control of ROS signaling might contribute to the classical distinctions between these two mature B cell subsets.

### Deletion of *Gclc* results in a drastic decrease of splenic MZB

**Fig. 2:**
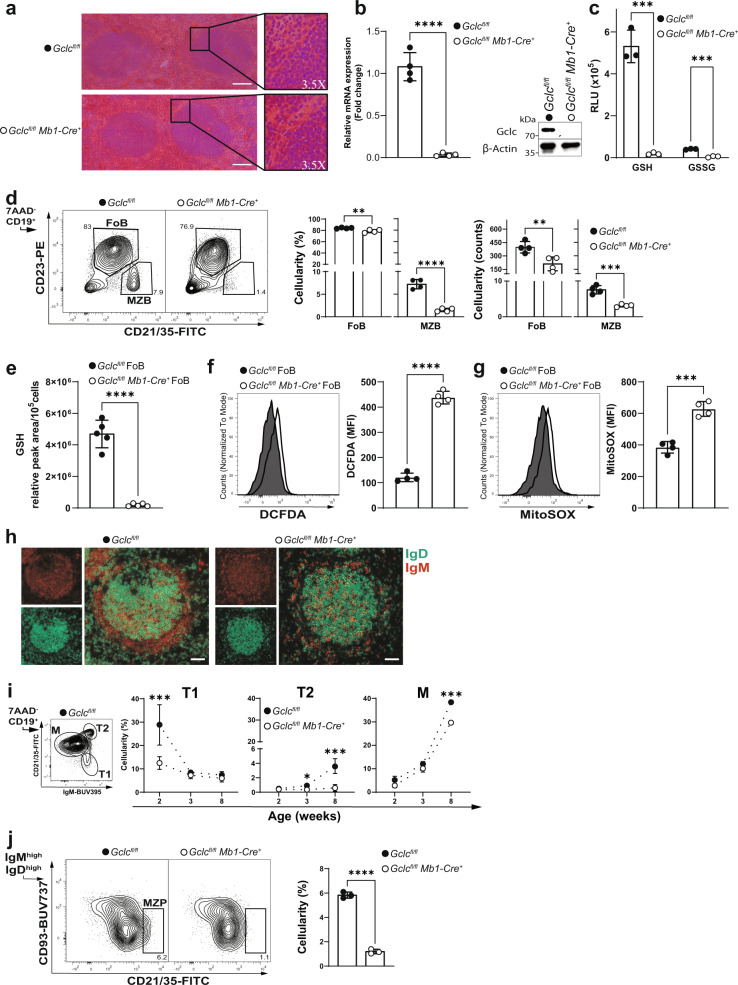
*Gclc* depletion induces a stark reduction in MZB. **a** Histology of spleen follicles resected from one *Gclc*^*fl/fl*^ and one *Gclc*^*fl/fl*^ *Mb1-Cre*^*+*^ mouse and stained with haematoxylin and eosin. Scale bars, 50 µm. Insert digital magnification, 3.5×. **b** Left: RT-qPCR of *Gclc* mRNA of total splenic B cells isolated from *Gclc*^*fl/fl*^ and *Gclc*^*fl/fl*^ *Mb1-Cre*^*+*^ mice (*n* = 4 animals examined over four independent experiments). Right: Representative immunoblot of Gclc protein in total splenic B cells isolated from spleen of *Gclc*^*fl/fl*^ and *Gclc*^*fl/fl*^ *Mb1-Cre*^*+*^ mice (*n* = 4 animals examined over three independent experiments). **c** Luminescence-based quantitation of intracellular GSH and GSSG in total splenic B cells isolated from *Gclc*^*fl/fl*^ and *Gclc*^*fl/fl*^ *Mb1-Cre*^*+*^ mice (*n* = 3 animals examined over three independent experiments). **d** Representative contour plot (left), percentages (middle) and numbers (right) statistic of splenic FoB and MZB from *Gclc*^*fl/fl*^ and *Gclc*^*fl/fl*^ *Mb1-Cre*^*+*^ mice (gated as in Supplementary Fig. 1a) (*n* = 4 animals examined over five independent experiments). **e** Relative quantitation via LC-MS of GSH from resting FoB of *Gclc*^*fl/fl*^ and *Gclc*^*fl/fl*^ *Mb1-Cre*^*+*^ mice (*n* = 5 animals examined over two independent experiments). Representative histogram and quantitation of DCFDA (**f**) and MitoSOX (**g**) staining for intracellular ROS and mitochondrial (mt) ROS detection in splenic FoB from *Gclc*^*fl/fl*^ and *Gclc*^*fl/fl*^ *Mb1-Cre*^*+*^ mice (gated as in Supplementary Fig. 1a) (*n* = 4 animals examined over three independent experiments). **h** Representative immunofluorescence staining to detect IgD (green) and IgM (red) in a single spleen follicle resected from one *Gclc*^*fl/fl*^ and one *Gclc*^*fl/fl*^ *Mb1-Cre*^*+*^ mouse. Scale bars: 50 µm. **i** Representative contour plot of splenic *Gclc*^*fl/fl*^ M, T1, and T2 B cells used to determine the percentages over time (weeks) of the gated population from spleens of *Gclc*^*fl/fl*^ and *Gclc*^*fl/fl*^ *Mb1-Cre*^*+*^ mice (*n* = 2–3 animals examined over three independent experiments). **j** Representative contour plot (left) and percentages statistic (right) of splenic MZP from *Gclc*^*fl/fl*^ and *Gclc*^*fl/fl*^ *Mb1-Cre*^*+*^ mice (gated as in Supplementary Fig. 1a) (*n* = 3 animals examined over three independent experiments). For all applicable figure panels, data are mean ± SD and each dot represents one single mouse, except for (**f**) where each panel represents the mean of 2–3 mice. In contour plots, numbers represent percentages of the cells gated. Significance (*P*) was calculated with unpaired t-test, except for (**c**) (2way ANOVA). *P ≤ 0.05; **P ≤ 0.01; ***P ≤ 0.001; ****P ≤ 0.0001.

We next examined the macroscopic structure of splenic B cell follicles and the distribution of FoB and MZB in control *Gclc*^*fl/fl*^ and mutant *Gclc*^*fl/fl*^ *Mb1-Cre*^*+*^ mice. At steady-state, histological examination did not reveal major macroscopic anomalies in the follicle architecture of mutant animals (Fig. 2a). However, follicles from *Gclc*^*fl/fl*^ *Mb1-Cre*^*+*^ mice showed partial loss of cellularity around the edges (Fig. 2a, insert). Ablation of *Gclc* in total splenic B cells was confirmed at both the mRNA and protein levels (Fig. 2b). Importantly, deletion of *Gclc* blocked reduced glutathione (GSH) synthesis, and, therefore, oxidized glutathione (GSSG) abundance was minimal in total B cells (Fig. 2c). This confirmed the efficient *Cre*-mediated deletion of the *Gclc* and also suggested that steady state B cells do not import GSH from the environment. As a consequence of GSH-deficiency, total B cells showed higher cytosolic and mitochondrial ROS (Supplementary Fig. 2a, b), although they were decreased in frequency and numbers (Supplementary Fig. 2c).

Comparative flow cytometric analysis of MZB (CD19^+^ CD23^low^ CD21/35^high^) and FoB (CD19^+^ CD23^high^ CD21/35^low^) in *Gclc*^*fl/fl*^ *Mb1-Cre*^*+*^ and control mice showed that *Gclc* deletion caused a stark decrease in MZB but not in FoB (Fig. 2d). However, in *Gclc-*deficient FoB no GSH was detectable and these cells accumulated ROS (Fig. 2e–g). Muri et al. have shown that MZB display increased sensitivity to lipid peroxidation in *Gpx4*-deficient mice^33^. Gpx4 uses GSH as cofactor and is needed to prevent lipid peroxidation, which depends on ROS. In line, lipid ROS accumulated in *Gclc*-deficient cells at steady state (Supplementary Fig. 2d).

Analysis of alternative MZB-specific surface proteins (i.e. IgM, CD1d, and CD24)^4,7^ (Supplementary Fig. 2e) and immunofluorescence microscopy (Fig. 2h) confirmed the lack of MZB in *Gclc*^*fl/fl*^ *Mb1-Cre*^*+*^ mice. Importantly, flow cytometric analysis of MZB both excluded any gene-dosage effect of *Gclc* expression and confirmed that loss of MZB was independent of any *Cre*-associated toxicity (Supplementary Fig. 2f, g), a concern raised in a previous study^52^. In line with the greater baseline expression of *Gclc* in B6 MZB compared to B6 FoB (Fig. 1a), these data suggest that GSH is required for the homeostatic persistence of MZB in the spleen.

### MZB differentiation is blocked in *Gclc*-deficient mice

The loss of MZB in *Gclc*^*fl/fl*^ *Mb1-Cre*^*+*^ mice prompted us to investigate the possibility of defects in the trafficking of MZB in the spleen. To this extent, previous reports have shown that the control of MZB localization in the spleen depends on signals delivered by integrins and chemokines such as ICAM-1 and VCAM-1^16,53^. Moreover, MZB entry and retention in the splenic marginal zone are tightly regulated by chemotactic molecules, such as C-X-C Motif Chemokine Ligand 13 (CXCL13) and sphingsoine-1-phosphate (S1P)^54^. However, the expression level of ICAM-1 and VCAM-1 in total splenocytes and serum levels of chemotactic factors (i.e. CXCL13 and S1P) were unchanged in *Gclc*-deficient mice when compared to control littermates (Supplementary Fig. 3a, b). Furthermore, it has been shown that trafficking and retention of MZB require specific macrophage-B cell interactions^53,55–57^. However, both Siglec-1^+^ marginal zone and MARCO^+^ metallophilic macrophages (MZM and MMM)^58^ formed similar structures in the spleen of *Gclc*^*fl/fl*^ *Mb1-Cre*^*+*^ mice compared to controls (Supplementary Fig. 3c). These data indicated that the factors responsible for MZB positioning in the marginal zone were not affected in *Gclc*-deficient mice.

To assess whether *Gclc* interferes with the development of MZB, which generally populate the spleen 2–3 weeks after birth^59^, we investigated the distribution of discrete maturation steps in the spleen. In line with an earlier study^60^, we identified three distinct B cell splenic maturation stages using CD21/35 and IgM (Fig. 2i, left): immature type 1 (T1), type 2 (T2), and mature (M) B cells. MZB cells are thought to develop from M B cells but also from T2 B cells^60,61^. We found that *Gclc* ablation prevented the formation of T1 B cells by 2 weeks of age, indicating that a block in the B cell differentiation process occurred at the very early stage in the spleen (Fig. 2i). Accordingly, T2 B cells did not develop in *Gclc*-deficient hosts (Fig. 2i, right). By 8 weeks, M B cells frequency dropped significantly, indicating that *Gclc*-deficient T1 B cells limit M B cells development and that *Gclc* is crucial for the transition from T1 to T2 B cells. Importantly, the lack of T2 B cells indicated that *Gclc* is needed in the transition to MZB or their precursors. To this extent, we studied the distribution of MZB precursors (MZP) in adult mice. As expected, *Gclc*-deficient mice showed lower frequencies of MZP compared to control mice (Fig. 2j), corroborating the data shown in Fig. 2i. Therefore, we concluded that defective B cell development causes the decrease of MZB content in *Gclc*^*fl/fl*^ *Mb1-Cre*^*+*^ mice.

Additionally, later studies have defined three subsets of CD93^+^ immature or “transitional” B cells (TrB1-3) in the spleen of adult mice by the expression of CD23 and IgM^62^. Using this phenotypical characterization, most of FoB and MZB are thought to derive from TrB1 that have transitioned to TrB2^61,62^. Thus, to better understand at which stage GSH is essential in the maintenance of B cells in the adult mouse, we measured *Gclc* expression and total GSH in TrB from mutant and control mice. *Gclc* was efficiently deleted in TrB cells (Supplementary Fig. 3d). Of note, wild-type TrB showed intermediate levels of *Gclc* mRNA compared to FoB and in MZB (Supplementary Fig. 3e), implying that *Gclc* expression might characterize distinct stages of splenic B cell development. Thus, we measured TrB1-3 distribution in B cell-specific *Gclc*-deficient mice and found that TrB1 were increased in *Gclc*-deficient mice compared to controls (Supplementary Fig. 3f), despite showing low GSH content (Supplementary Fig. 3g). A possible scenario is that *Gclc* deficiency causes a mild block at the TrB1 stage, possibly impacting the maturation of MZB precursors. Therefore, these data support the existence of regulatory functions of *Gclc* in the FoB-MZB fate decision.

Overall, the results shown above confirmed that migratory inputs are not affected by the absence of *Gclc* in B cells, and that failure to detect MZB in B cell-specific *Gclc*-deficient mice is not due to altered environmental cues. Instead, we have shown that in vivo ablation of *Gclc* in B cells caused a block in B cell differentiation, which results in the absence of MZP. This implies that the difference in redox flexibility between FoB and MZB precursors is necessary to allow B cell differentiation.

### The mTOR pathway confers MZB-like properties to GSH-deficient FoB

To investigate further the function of GSH in total splenic B cells, we performed a comparative single-cell CITE-seq analysis on B cells from *Gclc*^*fl/fl*^ and *Gclc*^*fl/fl*^ *Mb1-Cre*^*+*^ mice. tSNE visualization of the total B cell transcriptomic landscape revealed minor changes in the distribution of single-cell transcriptomes in the absence of *Gclc* (Supplementary Fig. 4a). Mirroring our flow cytometric analyses (Fig. 2d and Supplementary Fig. 2e), ADT signals and the SCINA assignments confirmed the loss of MZB in *Gclc*^*fl/fl*^ *Mb1-Cre*^*+*^ mice (Supplementary Fig. 4b, c). As expected, FoB were clearly detectable in *Gclc*^*fl/fl*^ *Mb1-Cre*^*+*^ mice by the expression of CD23-ADT and IgD-ADT (Supplementary Fig. 4c). To investigate the transcriptomic changes induced by *Gclc* deletion, we performed downstream analyses of FoB (Supplementary Fig. 4d). As shown in Supplementary Fig. 4e, PCA analysis indicated that principal component 1 (PC1) covered most of the variation in gene expression (58% of total variance), likely including the effect caused by *Gclc* deletion. Analysis of total gene transcripts per cell further suggested that the deletion of *Gclc*, and therefore the lack of GSH (Fig. 2e), influenced gene expression levels in FoB (Supplementary Fig. 4f).

**Fig. 3:**
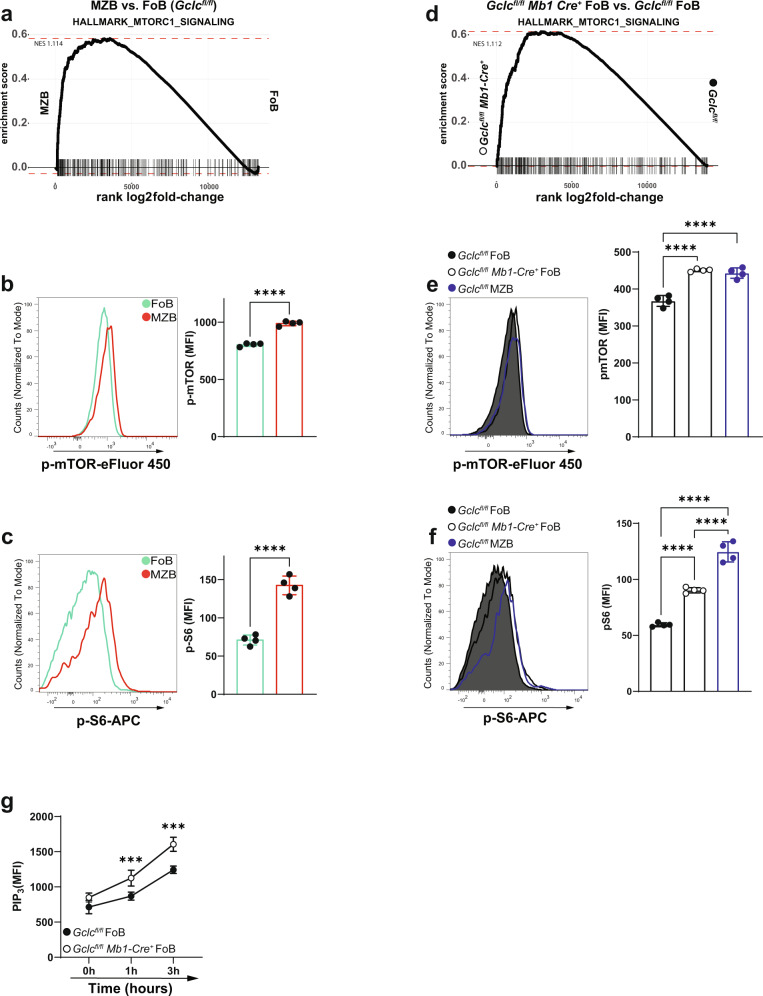
mTORC1 is comparably elevated in *Gclc*-sufficient MZB and *Gclc*-deficient FoB. **a** GSEA plot comparing expression of mTORC1 signaling hallmark gene sets between *Gclc*^*fl/fl*^ FoB and *Gclc*^*fl/fl*^ MZB. Representative histogram (left) and quantitation (right) of immunostaining to detect p-mTOR (**b**) and p-S6 (**c**) in B6 FoB and MZB (gated as in Supplementary Fig. 1a) (*n* = 4 animals examined over three independent experiments). **d** GSEA plot comparing the expression of mTORC1 signaling hallmark gene sets between *Gclc*^*fl/fl*^ and *Gclc*^*fl/fl*^ *Mb1-Cre*^*+*^ FoB. Representative histogram (left) and quantitation (right) of immunostaining to detect p-mTOR (**e**) and p-S6 (**f**) in *Gclc*^*fl/fl*^ and *Gclc*^*fl/fl*^ *Mb1-Cre*^*+*^ FoB, including *Gclc*^*fl/fl*^ MZB as control (gated as in Supplementary Fig. 1a) (*n* = 4 animals examined over four independent experiments). **g** Flow-cytometry measurements kinetic of PIP_3_ in sorted FoB from spleens of *Gclc*^*fl/fl*^ and *Gclc*^*fl/fl*^ *Mb1-Cre*^*+*^ at the indicated time points after stimulation with anti-IgM (*n* = 3 animals examined over two independent experiments). Data are mean ± SD. Significance (*P*) was calculated with unpaired t-test, except for **e**, **f** (one-way ANOVA), and **g** (2 way ANOVA). ***P ≤ 0.001; ****P ≤ 0.0001.

As noted above, mTOR signaling has emerged as a key control point for B cell functions^35^. As already shown, GSH-deficiency in B cells resulted in the absence of MZB but not of FoB (Fig. 2d, h). This prevented the direct analysis of *Gclc*-deficient MZB. This implies the existence of a GSH-dependent process that is more important for MZB than for FoB. To elucidate these regulation circuits and to investigate whether mTOR signaling might be involved in this abnormality, we performed gene set enrichment analysis (GSEA) on differentially expressed genes of *Gclc*-sufficient MZB vs. FoB (*Gclc*^*fl/fl*^ MZB vs. *Gclc*^*fl/fl*^ FoB) and of *Gclc*-deficient FoB vs. control FoB (*Gclc*^*fl/fl*^ *Mb1-cre*^*+*^ FoB vs. *Gclc*^*fl/fl*^ FoB). In accordance with previous reports^36,37^, our GSEA analysis revealed positive enrichment for genes of the mTORC1 signaling pathway in *Gclc*-sufficient MZB compared to FoB (Fig. 3a). Indeed, flow cytometric evaluation of resting B6 MZB confirmed increased phosphorylation of both mTOR and its canonical target S6 (Fig. 3b, c). Strikingly, our GSEA analysis also showed that ablation of *Gclc* in FoB displayed a similar increase in mTORC1 signaling genes compared to *Gclc*^*fl/fl*^ FoB and *Gclc*^*fl/fl*^ MZB (Fig. 3d). Corresponding increases in phosphorylated mTOR and S6 were detected in mutant FoB compared to control FoB (Fig. 3e, f and Supplementary Fig. 5a). These data pointed to an unexpected similarity between *Gclc*-deficient FoB and *Gclc*-sufficient MZB.

mTOR is also an important regulator of translation^63^, and supports an enhanced secretory apparatus and superior secretory activity of MZB compared to FoB^7,14^. Accordingly, we found that B6 MZB had a higher endoplasmic reticulum (ER)/Golgi ratio than B6 FoB as measured by their greater staining intensity of ER-tracker and giantin (Supplementary Fig. 5b), as well as by their increased phosphorylation of the translation initiation factor eIF4E (Supplementary Fig. 5c). In addition, our differential expression analysis identified upregulation of a considerable number of transcripts related to ribosomal proteins in *Gclc*^*fl/fl*^ MZB compared to *Gclc*^*fl/fl*^ FoB (Supplementary Fig. 5d). Additionally, gene ontology analysis detected accumulation of transcripts related to the three steps of protein synthesis (initiation, elongation, termination) in *Gclc*^*fl/fl*^ MZB compared to *Gclc*^*fl/fl*^ FoB (Supplementary Fig. 5e). To confirm this heightened protein translation in vitro, B6 FoB and B6 MZB were activated with anti-IgM, CD40 ligand and IL-4 or LPS, pulsed with puromycin, and chased for up to 6 h^64^. B6 MZB indeed showed greater protein synthesis compared to B6 FoB over time (Supplementary Fig. 5f). Strikingly, the absence of *Gclc* in FoB resulted in secretory properties that recapitulated those of *Gclc*-sufficient MZB at both the transcriptional and protein levels (Supplementary Fig. 5g–k). These findings confirmed a certain degree of similarity between *Gclc*-deficient FoB and *Gclc*-sufficient MZB, which was further substantiated by comparing differentially expressed genes between the two groups *Gclc*^*fl/fl*^ MZB vs. FoB and *Gclc*^*fl/fl*^ *Mb1-Cre*^*+*^ vs. control FoB (Supplementary Fig. 5l). This suggests that MZB and FoB depend on a different redox state and that the loss of GSH in FoB may induce MZB-like properties.

mTORC1 is regulated through the phosphatidylinositol 3 kinase (PI3K)/Protein kinase B (Akt)-dependent pathway^65^. In particular, the PI3K/AKT axis converts phosphatidylinositol-4,5-bisphosphate (PIP_2_) to phosphatidylinositol 3,4,5-trisphosphate (PIP_3_). A key repressor of this signaling, phosphatase and tensin homolog deleted on chromosome 10 (PTEN), dephosphorylates PIP_3_ to PIP_2_ regulating the activity of PI3K/AKT^66^. Time course in vitro activation showed that PIP_3_ levels were higher in GSH-deficient FoB compared to control cells (Fig. 3g). This suggested that the PI3K/AKT pathway is increased in *Gclc*-deficient FoB, and in turn can sustain increased mTORC1 activity. Importantly, *Gclc*-deficient FoB accumulate ROS (Fig. 2f, g), which are known inducers of PI3K/AKT^67,68^ and suppressors of PTEN^69^. Therefore, it is possible that the increased mTORC1 activation measured in FoB upon *Gclc* deletion can be a consequence of ROS accumulation and activation of the PI3K/AKT axis.

Collectively, these data might indicate that the diminished ROS buffering of GSH-deficient cells leads to an increased activation of the mTOR pathway which could drive to the MZB-like properties of *Gclc*-deficient FoB. We were thus prompted to explore the intriguing hypothesis that GSH-dependent control of ROS could play a role in defining the distinctive metabolic signaling of MZB and FoB.

### *Gclc* deletion enhances glycolytic reactions and glucose uptake in FoB

**Fig. 4:**
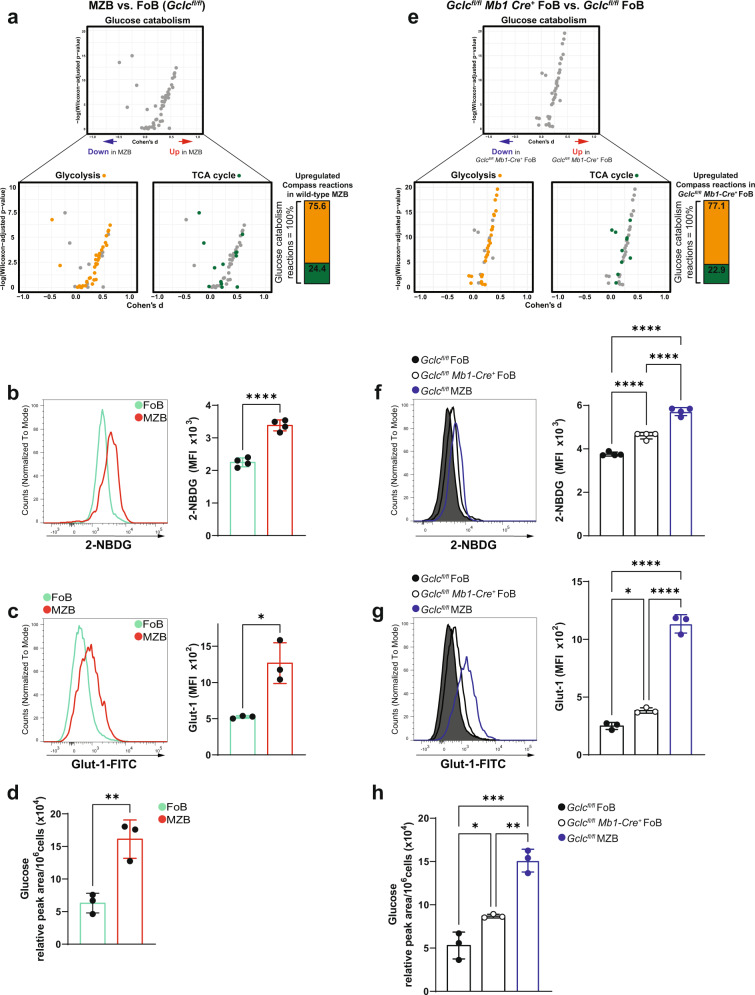
*Gclc*-deficient FoB show increased glycolytic reactions and glucose uptake, mirroring *Gclc*-sufficient MZB. **a** Compass analysis to compare glucose catabolism (top) and its components (glycolysis and TCA cycle) (bottom) in *Gclc*^*fl/fl*^ MZB vs. FoB. Each dot represents a single biochemical reaction. Means difference (Cohen’s d) and Wilcoxon rank sum *p* values were computed as described previously^70,71^. Vertical bar shows relative contributions (percentages) of upregulated glycolytic reactions and TCA reactions to total glucose catabolism computed from glycolysis and TCA cycle reactions in *Gclc*^*fl/fl*^ MZB vs. FoB. (**b**-**c**) Representative histogram (left) and quantitation (right) of 2-NBDG (**b**) and Glut-1 (**c**) staining in resting splenic B6 FoB and MZB (gated as in Supplementary Fig. 1a) (*n* = 3 animals examined over 3–4 independent experiments). **d** Quantitation of intracellular glucose level measured by LC-MS in resting *Gclc*-sufficient FoB and MZB (*n* = 3 animals examined over two independent experiments). **e**–**h** Same analyses as shown in (**a**–**d**), but comparing *Gclc*^*fl/fl*^ versus *Gclc*^*fl/fl*^ *Mb1-Cre*^*+*^ FoB and including *Gclc*^*fl/fl*^ MZB as control (gated as in Supplementary Fig. 1a) (*n* = 3–4 animals examined over 2–3 independent experiments). For all applicable figure panels, data are mean ± SD and each dot represents one single metabolic reaction or mouse. Significance (*P*) was calculated with unpaired t-test, with exception of (**f**)–(**h**) (one-way ANOVA). *P ≤ 0.05; **P ≤ 0.01; ***P ≤ 0.001; ****P ≤ 0.0001.

mTORC1 signaling plays a role in maturation and activation of both MZB and FoB^36–39^, and is key for the regulation of various metabolic pathways^34^. Therefore, in order to contextualize the complexity of the metabolic network and the impact of *Gclc* on it, we analyzed our transcriptomic data using Compass, a flux balance analysis-based approach that allows the characterization and interpretation of metabolic diversity at the single cell level^70,71^. Using this computational prediction method, we found that *Gclc*^*fl/fl*^ MZB exhibited increased glucose catabolism compared to *Gclc*^*fl/fl*^ FoB (Fig. 4a, top and Supplementary Data 2). Glucose can be catabolized to lactate during glycolysis or glucose-derived carbons can enter the tricarboxylic acid cycle (TCA). We, therefore, sorted the metabolic reactions identified by the Compass algorithm into two groups: glycolysis and TCA. This analysis indicated that glycolysis was upregulated in *Gclc*^*fl/fl*^ MZB compared to *Gclc*^*fl/fl*^ FoB (Fig. 4a, bottom and Supplementary Data 2), suggesting that MZB rely on glycolysis rather than oxidative metabolism at steady-state. To confirm these predictions, we measured glucose uptake with 2-(N-(7-Nitrobenz-2-oxa-1,3-diazol-4-yl)Amino)-2-Deoxyglucose (2-NBDG)^72^ in B6 MZB and FoB. In line with our Compass results, B6 MZB showed increased 2-NBDG uptake compared to B6 FoB (Fig. 4b) as well as higher expression of the glucose transporter Glut-1 as measured by flow cytometry (Fig. 4c). Moreover, total intracellular glucose measured by mass spectrometry was elevated in freshly isolated MZB compared to FoB (Fig. 4d), although the size difference between FoB and MZB^7^ might influence total glucose level.

Our data above indicated that GSH deficiency upregulated mTORC1 signaling in mutant FoB. Therefore, we sought to determine whether this heightened mTORC1 skewed FoB metabolism towards glycolysis. Indeed, our Compass analysis reflected a global increase in glucose catabolism in FoB when *Gclc* was ablated (Fig. 4e and Supplementary Data 3), just as occurred in *Gclc*-sufficient MZB (Fig. 4a). Mutant FoB also showed increased 2-NBDG uptake at steady-state (Fig. 4f), higher Glut-1 expression (Fig. 4g), and accumulation of glucose (Fig. 4h) compared to *Gclc*^*fl/fl*^ FoB and MZB. These data indicate that mutant FoB undergo an increase in oxidative state (i.e. low GSH and high ROS) that might induce some metabolic traits similar to those observed in *Gclc*-sufficient MZB.

### GSH-deficient FoB upregulate glycolysis but accumulate defective mitochondria

**Fig. 5:**
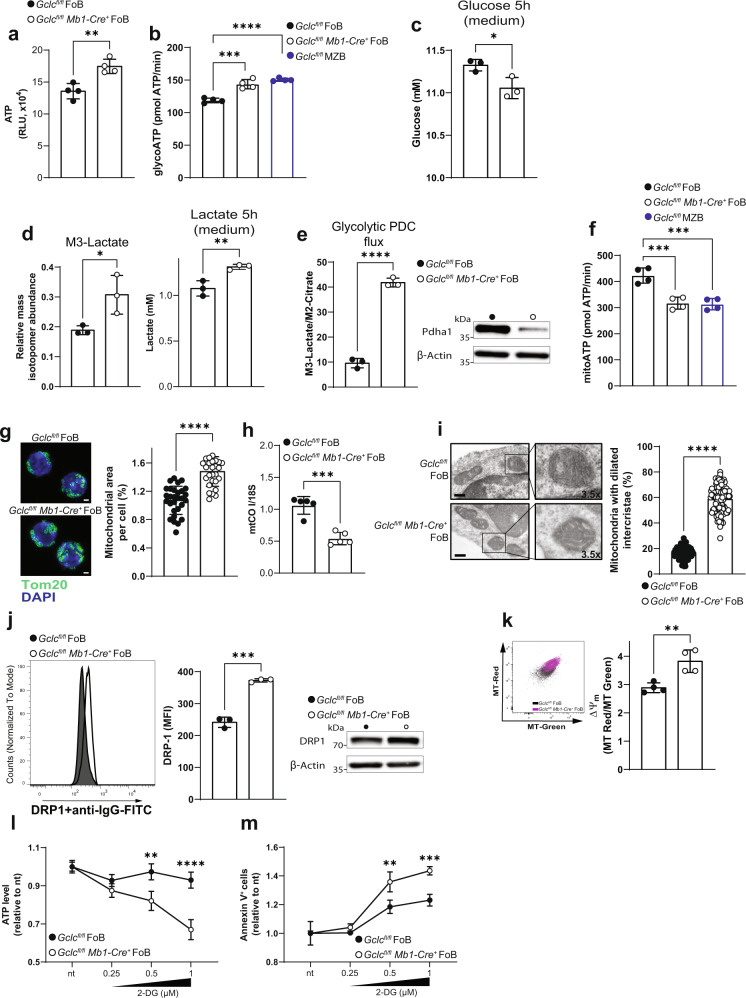
GSH deficiency increases glucose flux through glycolysis and leads to mitochondria accumulation in FoB. **a** Total ATP levels expressed in relative luminescence unit (RLU) in resting *Gclc*^*fl/fl*^ and *Gclc*^*fl/fl*^ *Mb1-Cre*^*+*^ FoB (*n* = 4 animals examined over 4 independent experiments). **b** Seahorse quantitation of glycolysis-derived ATP in resting *Gclc*^*fl/fl*^ and *Gclc*^*fl/fl*^ *Mb1-Cre*^*+*^ FoB and resting *Gclc*^*fl/fl*^ MZB (*n* = 3 animals examined over three independent experiments). **c** Quantitation of total glucose in culture supernatants of *Gclc*^*fl/fl*^ and *Gclc*^*fl/fl*^ *Mb1-Cre*^*+*^ FoB at 5 h post-stimulation with anti-IgM, CD40 ligand, and IL-4 (*n* = 3 animals examined over two independent experiments). **d** Left: Mass isotopomeric distribution (MID) of M3-lactate in *Gclc*^*fl/fl*^ and *Gclc*^*fl/fl*^ *Mb1-Cre*^*+*^ FoB that were incubated with ^13^C-glucose and assayed at 5 h post-activation with anti-IgM, CD40 ligand and IL-4. Right: quantitation of total lactate in culture supernatants of the cells in the left panel (*n* = 3 animals examined over three independent experiments). **e** Left: ratio of M3-lactate/M2-citrate in *Gclc*^*fl/fl*^ and *Gclc*^*fl/fl*^ *Mb1-Cre*^*+*^ FoB that were incubated with ^13^C-glucose and assayed at 5 h post-activation. Right: representative blot of Pdha1 protein from total cell lysis of resting *Gclc*^*fl/fl*^ and *Gclc*^*fl/fl*^ *Mb1-Cre*^*+*^ FoB (*n* = 3 animals examined over three independent experiments). **f** Seahorse quantitation of mitochondria-derived ATP in resting *Gclc*^*fl/fl*^ and *Gclc*^*fl/fl*^ *Mb1-Cre*^*+*^ FoB and *Gclc*^*fl/fl*^ MZB (*n* = 4 animals examined over three independent experiments). **g** Left: Representative confocal microscopic image of mitochondrial morphology in resting *Gclc*^*fl/fl*^ and *Gclc*^*fl/fl*^ *Mb1-Cre*^*+*^ FoB. Mitochondria are green (Tom20) and nuclei are blue (DAPI). Scale bars, 1 µm; Right: Quantitation of mitochondrial area (Tom20 area) per cell. Each dot represents a single cell (*n* = 2 animals examined over three independent experiments). **h** Mitochondrial mass quantitation [ratio between mitochondrial cytochrome c oxidase subunit I (CO I) and nuclear 18S ribosomal RNA] as determined by RT-qPCR of DNA from *Gclc*^*fl/fl*^ and *Gclc*^*fl/fl*^ *Mb1-Cre*^*+*^ FoB (*n* = 5 animals examined over three independent experiments). **i** Left: representative TEM image of ultrastructural mitochondrial morphology in *Gclc*^*fl/fl*^ and *Gclc*^*fl/fl*^ *Mb1-Cre*^*+*^ FoB. Scale bars, 300 nm. Insert digital magnification, 3.5×. Right: percentage of mitochondria with enlarged intercristae counted from 25 cells/genotype (*n* = 3 animals examined over 2 independent experiments). Each dot represents a single mitochondrium. **j** Representative flow-cytometry histogram (left) and quantitation (middle), and blot (right) of DRP1 in resting splenic *Gclc*^*fl/fl*^ and *Gclc*^*fl/fl*^ *Mb1-Cre*^*+*^ FoB (gated as in Supplementary Fig. 1a) (*n* = 3 animals examined over two independent experiments). **k** Representative contour plot (left) and quantitation of derived ΔΨ_m_ values (right) of Mitotracker dyes in *Gclc*^*fl/fl*^ and *Gclc*^*fl/fl*^ *Mb1-Cre*^*+*^ FoB (gated as in Supplementary Fig. 1a) (*n* = 4 animals examined over three independent experiments). (**l**–**m**) Normalized ATP levels (RLU) (**l**) and percentage of Annexin V-expressing cells (**m**), in *Gclc*^*fl/fl*^ and *Gclc*^*fl/fl*^ *Mb1-Cre*^*+*^ FoB after 5 h incubation with the indicated concentrations of 2-DG (*n* = 3 animals examined over two independent experiments). For all applicable figure panels, data are mean ± SD and each dot represents one single mouse, except for (**l**) and (**m**) where each dot represents the mean of triplicates. Significance (*P*) was calculated with unpaired t-test, except for **b**, **f** (one-way ANOVA), and **l**, **m** (2 way ANOVA). *P ≤ 0.05; **P ≤ 0.01; ***P ≤ 0.001; ****P ≤ 0.0001. MFI: mean fluorescence intensity.

Based on our scRNA analyses and the effect of *Gclc* deficiency on glucose uptake (Fig. 4e–h), we compared ATP levels in *Gclc*^*fl/fl*^ and *Gclc*^*fl/fl*^ *Mb1-Cre*^*+*^ FoB prior to activation. Interestingly, we detected increased total ATP in *Gclc*-deficient FoB (Fig. 5a). This finding was confirmed by flux analysis (Fig. 5b) and further suggests an increased glycolytic activity of *Gclc*-deficient FoB, a dependence that is not observed in *Gclc*-deficient T cells^43^. Importantly, we also confirmed that *Gclc*^*fl/fl*^ MZB and B6 MZB showed increased glycolysis-derived ATP (Fig. 5b and Supplementary Fig. 6a). These data further confirm our Compass predictions (Fig. 4a, e) and strengthen the metabolic parallel between GSH-deficient FoB and GSH-sufficient MZB.

To further study the dynamics of glucose fluxes in B cell subsets, we measured glucose levels in the culture medium of *Gclc*^*fl/fl*^ and *Gclc*^*fl/fl*^ *Mb1-Cre*^*+*^ FoB after 5 h activation with anti-IgM, CD40 ligand and IL-4. We found that the activated mutant cells consumed more glucose than controls (Fig. 5c). Moreover, following ^13^C-glucose tracing in activated *Gclc*^*fl/fl*^ and *Gclc*^*fl/fl*^ *Mb1-Cre*^*+*^ FoB, we detected an increased contribution of glycolytic carbon to lactate within the mutant cells (Fig. 5d, left). In line, lactate also accumulated to a higher extent in the culture medium of *Gclc*^*fl/fl*^ *Mb1-Cre*^*+*^ FoB as compared to control cells (Fig. 5d, right). To further substantiate these results, we measured the expression of hexokinase 1 (HK1), a rate limiting enzyme of glycolysis. HK1 content increased upon *Gclc*-ablation in FoB and in *Gclc*^*fl/fl*^ MZB (Supplementary Fig. 6b). This was paralleled by the accumulation of glucose-6-phosphate (6P) in steady state GSH-deficient FoB (Supplementary Fig. 6c). Concomitantly, we measured increased pyruvate levels in mutant compared to control cells (Supplementary Fig. 6d), confirming increased glycolysis upon *Gclc*-ablation.

Furthermore, we determined the flux of glycolytic carbon through the pyruvate dehydrogenase complex (PDC) by stable-isotope labeling with ^13^C-glucose. Glycolysis converts the ^13^C-glucose tracer to M3-pyruvate isotopologues which are oxidized to M2-acetyl-CoA by PDC and finally condensed with oxaloacetate to M2-citrate. We found that the glycolytic flux into the TCA cycle was reduced in *Gclc*^*fl/fl*^ *Mb1-Cre*^*+*^ FoB compared to *Gclc*^*fl/fl*^ FoB, as indicated by increased M3-lactate and reduced M2-citrate (Supplementary Fig. 6e) and a higher M3-lactate/M2-citrate ratio in the mutant cells (Fig. 5e). In line, expression of the catalytic alpha subunit of PDC (Pdha-1) was decreased in *Gclc*-deficient cells (Fig. 5e), which provides a possible explanation for the reduced carbon flux into the TCA. Thus, blocking GSH synthesis in FoB preferentially promotes ATP production through aerobic glycolysis.

Most of the ATP in a cell is generated by the combined action of the TCA cycle and oxidative phosphorylation (OXPHOS) within the mitochondria^73,74^. Our data showed that loss of *Gclc* decreased glucose flux into the TCA cycle (Fig. 5e and Supplementary Fig. 6e), suggesting a disturbance in mitochondrial ATP generation. Indeed, flux analysis showed that, similarly to GSH-sufficient MZB, *Gclc*-deficient FoB at rest showed decreased amounts of mitochondria-derived ATP (Fig. 5f and Supplementary Fig. 6f), implying that *Gclc* deficiency alters mitochondrial metabolism in FoB. In turn, we speculated that this impairment of mitochondrial metabolism in FoB might explain the increase in aerobic glycolysis for ATP production.

To acquire more information about the mitochondrial function of *Gclc*-deficient FoB, we investigated mitochondrial size and conformation. Interestingly, the lack of mitochondrial GSH (mtGSH) in *Gclc*^*fl/fl*^ *Mb1-Cre*^*+*^ FoB (Supplementary Fig. 6g) resulted in an increased mitochondrial area compared to control FoB (Fig. 5g) as well as a lower mitochondrial DNA/nuclear DNA ratio as measured by quantitative PCR (Fig. 5h). Crucially, wild-type MZB are known to be prone to activation compared to FoB^7,8,75^, and we found that both *Gclc*^*fl/fl*^ *Mb1-Cre*^*+*^ FoB and B6 MZB showed a similar increase in mitochondrial mass (Fig. 5h and Supplementary Fig. 6h), further substantiating similarity. Thus, interference with GSH synthesis in FoB induced an increase in mitochondrial mass without concomitant mtDNA replication. Next, we visualized mitochondria of FoB by electron microscopy to examine the mitochondrial inner space and the conformation of mitochondrial cristae. This analysis confirmed the accumulation of fragmented mitochondria, which exhibited a dilated intercristae space, in *Gclc*-deficient FoB (Fig. 5i). This is in line with the higher expression of the major mitochondrial fission GTPase, dynamin-related protein 1 (DRP1)^76^ (Fig. 5j). Taken together, these data imply that loss of *Gclc* alters mitochondrial structure and metabolism of FoB.

To gain more insight into the effect of GSH paucity on mitochondria, we used MitoTracker (MT) green and MT red staining to assess mitochondrial membrane potential (ΔΨ_m_) with flow cytometry. We found that *Gclc* deficiency increased ΔΨ_m_ in mutant FoB compared to controls (Fig. 5k). However, loose mitochondrial cristae have been associated with poor electron transport chain (ETC) efficiency in T cells^77^, suggesting that other factors might be responsible for the increased |ΔΨ_m_| in mutant FoB. Coupled with our data, this observation suggests that GSH is critical for the steady state function of mitochondria, whose fitness is crucial for the maintenance of MZB. In FoB, we found that ATP levels in *Gclc*-deficient, but not in *Gclc*-sufficient FoB dropped in a dose-dependent fashion in response to inhibition of glycolysis with 2-deoxy-D-glucose (2-DG) or galactose (Fig. 5l and Supplementary Fig. 6i). In parallel, glycolysis inhibition increased cell death in *Gclc*-deficient FoB to a stronger extent when compared to controls (Fig. 5m and Supplementary Fig. 6j), confirming the dependency of GSH-deficient FoB on glycolysis.

Taken together, our data indicate that the loss of GSH triggered by *Gclc* deletion induces a defective mitochondrial accumulation in FoB, which may lead to compensation through upregulation of glycolytic metabolism. This observation implies the existence of a regulatory function for GSH in the context of energy metabolism shifts in B cells.

### Absence of *Gclc* impairs mitochondrial respiration in FoB

**Fig. 6:**
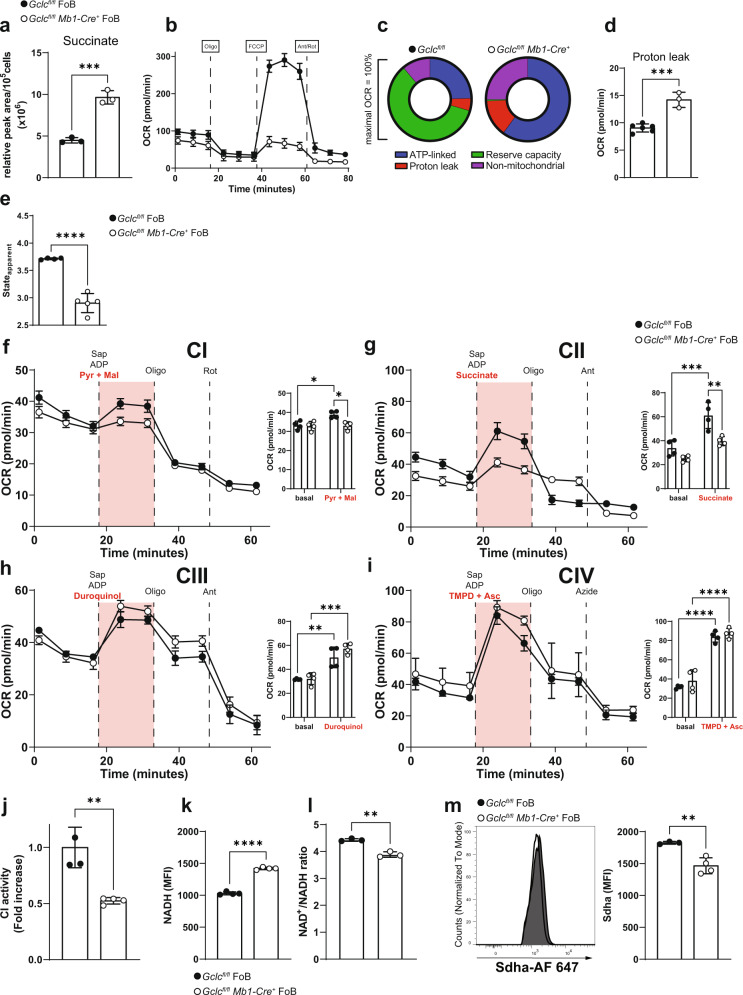
Mitochondrial respiratory complex I and II are dysfunctional in *Gclc*-deficient FoB. **a** Quantitation of intracellular succinate level measured by LC-MS in resting *Gclc*^*fl/fl*^ and *Gclc*^*fl/fl*^ *Mb1-Cre*^*+*^ FoB (*n* = 3 animals examined over two independent experiments). **b** Seahorse quantitation of OCR of resting *Gclc*^*fl/fl*^ and *Gclc*^*fl/fl*^ *Mb1-Cre*^*+*^ FoB at the indicated time points (*n* = 3–4 animals examined over 5 independent experiments). **c** OCR components from (**b**) plotted as proportions of maximal OCR (after FCCP treatment) (*n* = 3–4 animals examined over five independent experiments). **d** Quantitation of the contribution of proton leakage from (**b**) as determined by Ant/Rot treatment (*n* = 3–4 animals examined over five independent experiments). **e** Respiratory status of *Gclc*^*fl/fl*^ and *Gclc*^*fl/fl*^ *Mb1-Cre*^*+*^ FoB in (**b**) displayed as state_apparent_ (*n* = 3–4 animals examined over five independent experiments). **f**–**i** OCR of *Gclc*^*fl/fl*^ and *Gclc*^*fl/fl*^ *Mb1-Cre*^*+*^ FoB sequentially treated with saponin (Sap), adenosine diphosphate (ADP), and the indicated substrates (red) for ETC complexes CI (**f**), CII (**g**), CIII (**h**) and CIV (**i**), as indicated. Side bar graphs show basal and substrate-induced (red) OCR. Oligo: oligomycin A; Rot: rotenone; Ant: antimycin A; TMPD: N,N,N′,N′-Tetramethyl-p-phenylenediamine; Asc: ascorbate; Pyr: pyruvate; Mal: malate (*n* = 4 animals examined over two independent experiments). **j** Quantitation of relative activity of CI in *Gclc*^*fl/fl*^ and *Gclc*^*fl/fl*^ *Mb1-Cre*^*+*^ FoB as measured by colorimetric assay (*n* = 3–4 animals examined over two independent experiments). **k** Quantitation of NADH autofluorescence in *Gclc*^*fl/fl*^ and *Gclc*^*fl/fl*^ *Mb1-Cre*^*+*^ FoB (gated as in Supplementary Fig. 1a) as measured by FACS (*n* = 4 animals examined over three independent experiments). **l** CI activity in *Gclc*^*fl/fl*^ and *Gclc*^*fl/fl*^ *Mb1-Cre*^*+*^ FoB expressed as the ratio of NAD^+^ (CI substrate) to NADH (CI product), as measured by colorimetric assay (*n* = 3–4 animals examined over two independent experiments). **m** Representative histogram (left) and quantitation (right) of immunostaining to detect the expression of succinate dehydrogenase a (Sdha, the major subunit of CII) in *Gclc*^*fl/fl*^ and *Gclc*^*fl/fl*^ *Mb1-Cre*^*+*^ FoB (gated as in Supplementary Fig. 1a) (*n* = 3 animals examined over three independent experiments). For all applicable figure panels, data are mean ± SD and each dot represents one single mouse, except for (**b**) and (**f**)–(**i**) where each dot represents the mean of 3–5 mice. Significance (*P*) was calculated with unpaired t-test, except for **f**–**i** (2 way ANOVA). **P ≤ 0.01; ***P ≤ 0.001; ****P ≤ 0.0001.

Our data above showed that glucose-derived fluxes into the TCA cycle were reduced in GSH-deficient FoB. However, in our analyses of ^13^C-glucose, we found that total and labelled succinate accumulated to higher levels in mutant cells than in *Gclc*^*fl/fl*^ FoB (Fig. 6a and Supplementary Fig. 6k). Within the TCA cycle, succinate is converted into fumarate by the succinate dehydrogenase (SDH) which represents the respiratory ETC complex II (CII)^73^ (Supplementary Fig. 6l). Accordingly, SDH flux showed lower conversion into M2-fumarate in *Gclc*^*fl/fl*^ *Mb1-Cre*^*+*^ FoB compared to control cells (Supplementary Fig. 6m). Because SDH is the only enzyme that participates in both the TCA cycle and the ETC, we speculated that the ETC might be compromised in *Gclc*-deficient FoB, thus explaining the inhibition of SDH and the accumulation of succinate. Indeed, the basal oxygen consumption rate (OCR) of *Gclc*-deficient FoB measured by extracellular flux analysis was slightly reduced compared to *Gclc*^*fl/fl*^ FoB (Fig. 6b and Supplementary Fig. 6n), indicating that loss of GSH results in dysfunctional mitochondrial respiration in FoB at steady state. However, no difference in the OCR profiles of mutant and control FoB was detected upon treatment with oligomycin A (Fig. 6b and Supplementary Fig. 6o) which inhibits the ATP synthase, and thus, suggests that ADP phosphorylation capacity is unchanged. Interestingly, the most striking respiratory difference attributable to loss of GSH emerged upon treatment of *Gclc*^*fl/fl*^ and *Gclc*^*fl/fl*^ *Mb1-Cre*^*+*^ FoB with the mitochondrial ionophore FCCP, which increases the OCR to the maximal respiration (i.e. reserve capacity)^78^. Upon FCCP treatment, the ETC activity rate increases in the attempt to restore the proton gradient and re-couple it to OXPHOS. We found that an absence of *Gclc* in FoB prevented this FCCP-mediated uncoupling (Fig. 6b and Supplementary Fig. 6p), suggesting that GSH promotes electron transport through the ETC in FoB. However, transient treatment with GSH did not recover mitochondrial function (Supplementary Fig. 6q), possibly because of irreversible ROS-mediated modifications.

Previous work has established that any residual OCR detected after oligomycin A treatment is due to non-respiratory oxygen consumption, which is usually ascribed to proton leakage through the mitochondrial inner membrane^79^. We found that, despite the similar oligomycin A-dependent OCR profiles in *Gclc*^*fl/fl*^ and *Gclc*^*fl/fl*^ *Mb1-Cre*^*+*^ FoB (Fig. 6b and Supplementary Fig. 6o), the contributions to the maximal OCR value in each genotype by ATP-linked OCR*,* proton leakage, reserve capacity, and non-mitochondrial OCR were altered by *Gclc* deficiency (Fig. 6c). In particular, *Gclc*-deficient FoB experienced greater proton leakage (Fig. 6c, d), which together with increased ROS (Fig. 2f, g) might lead to damage of the mitochondrial membrane and/or ETC malfunction. In this regard, it is tempting to speculate that the accumulation of succinate (the CII substrate in the TCA cycle) in GSH-deficient FoB (Fig. 6a and Supplementary Fig. 6k) might be associated with the observed ETC insensitivity to FCCP treatment (Fig. 6b and Supplementary Fig. 6p).

Mitochondrial damage is often defined as a deviation of respiratory parameters or states, which were originally defined in vitro using isolated mitochondria^80–82^. However, these measurements can also be derived from measurements with whole cells (Supplementary Fig. 6r). In particular, state 3 respiration is equivalent to the OCR after FCCP treatment (i.e. maximal respiration induced by the substrate + ADP), and state 4 corresponds to the consumption rate upon oligomycin A addition (i.e. absence of substrate-dependent respiration)^83^. These assumptions allow for the calculation of the intermediate respiratory state or state_apparent_ (Supplementary Fig. 6s)^84,85^, which is an indicator of mitochondrial workload. From the assay performed in Fig. 6b, we determined a state_apparent_ value of 2.94 ± 0.15 in *Gclc-*deficient FoB (Fig. 6e), suggesting that FoB with *Gclc* deficiency experience a higher basal rate of mitochondrial activity compared to control cells. This higher activity results from the insensitivity of ETC to FCCP treatment and is consistent with the limited reserve capacity of *Gclc*-deficient FoB (Fig. 6b and Supplementary Fig. 6p). Taken together, these results establish that loss of GSH in FoB impairs mitochondrial respiration.

### Oxidative phosphorylation is disrupted at complex I and II in GSH-deficient follicular B cells

To determine whether the accumulation of succinate in *Gclc*-deficient FoB was caused by a dysfunctional ETC, we assessed the respiratory parameters of each ETC complex. First, we performed extracellular flux analysis to assay the mitochondrial complex-dependent OCR profile in saponin-permeabilized *Gclc*^*fl/fl*^ and *Gclc*^*fl/fl*^ *Mb1-Cre*^*+*^ FoB. Under normal conditions, CI regenerates the reducing equivalent NADH to NAD^+^ (Supplementary Fig. 6l). However, CI showed no activation upon substrate injection (pyruvate + malate) in *Gclc*-deficient FoB (Fig. 6f). Moreover, CII activation in *Gclc*^*fl/fl*^ *Mb1-Cre*^*+*^ FoB was also negligible compared to CII from control cells (Fig. 6g). Thus, both CI and CII exhibited a significant lower activity, which was confirmed by a lower state_apparent_ upon *Gclc* ablation (Supplementary Fig. 6t). However, substrate-dependent CIII and CIV OCR showed a tendency, although not significant, to be increased in GSH-deficient FoB cells compared to control FoB (Fig. 6h, i). CIII and CIV (together with CI) of the ETC act as H^+^ pump and build up the membrane potential. This suggests the possibility of a mild compensatory activation of CIII and CIV that may explain the higher intensity of MT red binding (i.e. higher ΔΨ_m_) in mutant FoB (Fig. 5k) despite the higher membrane leak (Fig. 6d). These data indicate that GSH-dependent ETC dysfunction mostly affected the activity of CI and CII. In line with these results, we detected lower CI activity in total lysates of *Gclc*-deficient FoB compared to control FoB (Fig. 6j). Consequently, NADH levels were increased in the mutant cells (Fig. 6k) due to reduced NAD^+^ regeneration which resulted in a lower NAD^+^/NADH ratio (Fig. 6l). Moreover, the expression level of the main subunit of CII (succinate dehydrogenase A, Sdha) was decreased in *Gclc*-deficient FoB (Fig. 6m). In line, ETC complex protein levels were reduced in mutant FoB compared to controls (Supplementary Fig. 6u). This included the CIII subunit core protein 2 and CIV subunit MTCO1, indicating that GSH-deficiency altered protein expression independently of the overall activity of CIII and CIV.

These data indicate that GSH plays a role in sustaining the activities of CI and CII, and so of the overall ETC. In particular, our energetic steady-state analyses (Fig. 6f and Supplementary Fig. 6t) show that loss of GSH induces a partial uncoupling of ETC from OXPHOS, suggesting that *Gclc* function has a direct effect on the metabolic dependencies of FoB. GSH deficiency has a large negative impact on the mitochondrial respiration machinery, slowing down the TCA cycle at the ETC branch point (i.e. CII). Thus, we have identified a previously unknown role of GSH in B cells: maintaining mitochondrial ETC activity.

### *Gclc* is required for T cell-independent and -dependent B cell immune responses

**Fig. 7:**
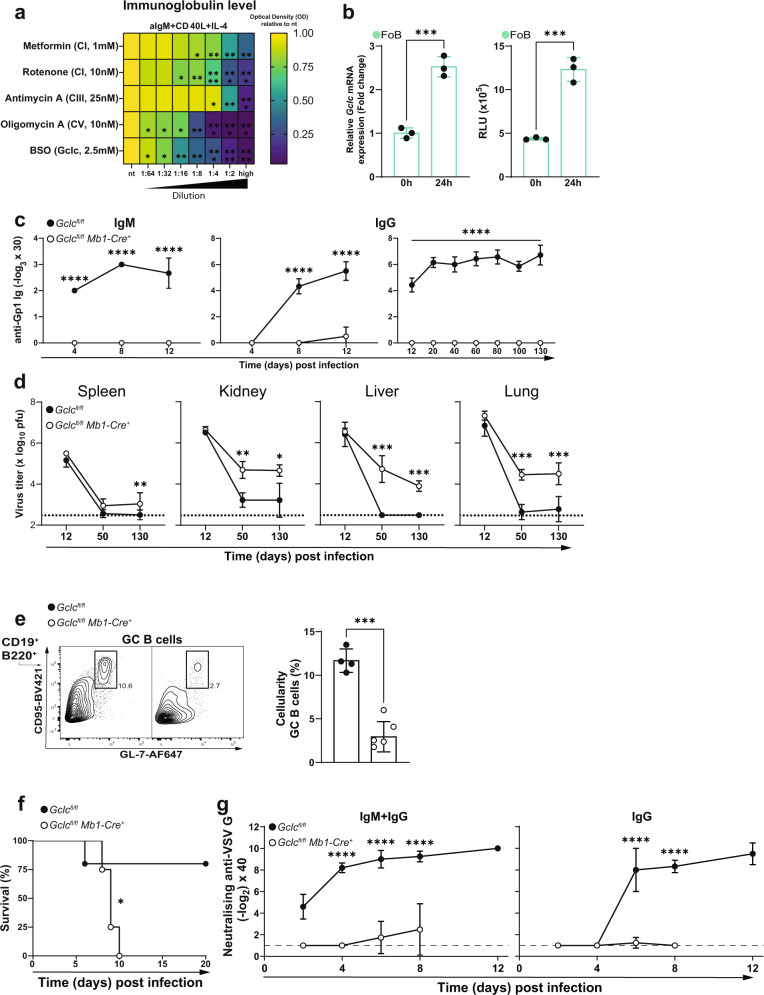
*Gclc* deficiency impairs B cell-mediated immune responses in vivo. **a** Heatmap showing relative expression of immunoglobulin level of 4d activated B6 FoB with increasing concentration of mitochondrial inhibitors and BSO. High concentration shown in brackets. Statistics are relative to nt. **b** Left: RT-qPCR of *Gclc* mRNA in B6 FoB at 0 or 24h after activation in vitro with anti-IgM, CD40 ligand and IL-4. Right: Luminescence-based quantitation of intracellular ROS in resting and 24h stimulated B6 FoB (*n* = 3 animals examined over two independent experiments). **c** Titers of IgM (left) and IgG (middle and right) in serum of LCMV Cl13-infected *Gclc*^*fl/fl*^ and *Gclc*^*fl/fl*^ *Mb1-Cre*^*+*^ mice as measured by ELISA at the indicated time points (*n* = 7 animals examined over 3–5 independent experiments). **d** LCMV Cl13 viral titers in the indicated organs of LCMV Cl13-infected *Gclc*^*fl/fl*^ and *Gclc*^*fl/fl*^ *Mb1-Cre*^*+*^ mice as measured by plaque assay at the indicated time points (*n* = 7 animals examined over 3–5 independent experiments). **e** Representative contour plot (left) and quantitation (right) of GC B cells (gated as in Supplementary Fig. 1a) in spleens of *Gclc*^*fl/fl*^ and *Gclc*^*fl/fl*^ *Mb1-Cre*^*+*^ mice as measured by FACS on day 12 post-LCMV Cl13 infection (*n* = 3 animals examined over two independent experiments). **f** Survival curve of *Gclc*^*fl/fl*^ and *Gclc*^*fl/fl*^ *Mb1-Cre*^*+*^ mice upon infection with VSV (*n* = 7 animals examined over two independent experiments). **g** Titers of neutralizing IgM and/or IgG antibodies against VSV-G protein in serum of *Gclc*^*fl/fl*^ and *Gclc*^*fl/fl*^ *Mb1-Cre*^*+*^ mice as measured by ELISA at the indicated time points (*n* = 7 animals examined over two independent experiments). Dotted/dashed lines represent the detection limit of the assay. For all applicable figure panels, data are mean ± SD and each dot represents one single mouse, except for (**a**), (**c**), (**d**), (**f**) and (**g**), where each panel or dot represents the mean of 3–7 mice. In contour plots, numbers represent percentages of the cells gated. Significance (*P*) was calculated with 2 way ANOVA, except for **b** and **e** (unpaired t-test) and **f** (log-rank test). *P ≤ 0.5; **P ≤ 0.01; ***P ≤ 0.001; ****P ≤ 0.0001.

Next, we aimed to understand how the metabolic perturbations caused by B cell-specific *Gclc* ablation impinge on immune responses and in particular how the impaired mitochondrial metabolism affects the antibody response. Therefore, we treated in vitro activated B6 FoB with various mitochondrial inhibitors and the Gclc inhibitor BSO and measured total immunoglobulins from the supernatants. After 4 days of activation with anti-IgM, CD40 ligand, and IL-4, we found that blocking of mitochondrial metabolism with mitochondrial inhibitors or blocking GSH synthesis by BSO had lowered antibody production of FoB in a dose-dependent manner (Fig. 7a). Thus, we reasoned that *Gclc* expression and GSH are needed upon activation of FoB. Indeed, levels of *Gclc* transcripts and reduced glutathione (GSH) increased upon activation B6 FoB in vitro (Fig. 7b), confirming our hypothesis.

Next, we studied B cell functions in vivo and measured antibody production. As expected, T cell-independent (TI) type II immunization with TNP-Ficoll to evaluate MZB-dependent antigen binding and early antibody production^86–88^ confirmed the deficit of MZB in *Gclc*^*fl/fl*^ *Mb1-Cre*^*+*^ mice (Supplementary Fig. 7a, b). The absence of MZB-mediated immunity was substantiated by the lack of anti-TNP IgM at 7 days post-immunization with TNP-Ficoll in *Gclc*^*fl/fl*^ *Mb1-Cre*^*+*^ mice (Supplementary Fig. 7c). Furthermore, to study the long-term antibody response and FoB function, we infected *Gclc*^*fl/fl*^ and *Gclc*^*fl/fl*^ *Mb1-Cre*^*+*^ mice with lymphocytic choriomeningitis virus Clone 13 (LCMV Cl13), which causes a chronic infection^89–93^. We monitored levels of antibodies against LCMV Cl13 glycoprotein 1 (Gp1) by ELISA at various time points after infection and observed that both early (IgM) and late (IgG) responses were undetectable in serum of infected mutant mice, in contrast to infected controls (Fig. 7c). Indeed, viremia in multiple tissues of infected *Gclc*^*fl/fl*^ *Mb1-Cre*^*+*^ animals was detected by 130 days post-infection (Fig. 7d), suggesting that *Gclc*^*fl/fl*^ *Mb1-Cre*^*+*^ animals are unable to efficiently control LCMV Cl13 replication.

The lack of LCMV-specific antibodies in our mutant mice could be caused by either a failure of the GC reaction or a defect in antibody production. To this extent, we detected a reduction in CD95^+^GL-7^+^ GC B cells in *Gclc*^*fl/fl*^ *Mb1-Cre*^*+*^ mice compared to controls (Fig. 7e) at 12 days post-infection. These data imply that the absence of *Gclc* prevents B cells from establishing GC reactions, and therefore, hinders antibody production.

LCMV is a non-cytopathic virus that replicates slowly, which favors its persistence due to the failure of the host to mount an efficient cytotoxic T cell response^89,94^. In order to exclude any virus-dependent effects, we further investigated the T cell-dependent (TD) response in *Gclc*^*fl/fl*^ *Mb1-Cre*^*+*^ mice using a stronger, T cell-driven model of cytopathic viral infection. Vesicular stomatitis virus (VSV) first induces the antiviral activity of endogenously-produced interferon and then the generation of neutralizing antibodies to the VSV-G glycoprotein, measures that are critical for the control of this virus^95,96^. We infected *Gclc*^*fl/fl*^ and *Gclc*^*fl/fl*^ *Mb1-Cre*^*+*^ mice with VSV and found that the mutants succumbed more rapidly to the infection than controls (Fig. 7f) and lacked neutralizing IgM and IgG antibodies against VSV-G glycoprotein (Fig. 7g). Accordingly, we found that in vitro activation and the costimulatory potential of GSH-deficient FoB was decreased compared to control cells upon 24h stimulation as indicated by lower expression of CD25, CD69, GL-7 and CD80/CD86 (Supplementary Fig. 7d). Consequently, low activation prevented proliferation in vitro (Supplementary Fig. 7e) and adds another layer that explains the lack of humoral response upon in vivo immunizations.

Collectively, these data confirm the functional absence of MZB in *Gclc*^*fl/fl*^ *Mb1-Cre*^*+*^ mice and demonstrate that *Gclc* is critical for FoB-mediated GC reactions and antibody-mediated immunity to infections. We have therefore established a link between a metabolic switch in response to GSH loss to the inability to mount antibody-mediated immune responses in vivo.

## Discussion

ROS-induced signals regulate B cell activation and early metabolic reprogramming^97,98^, and a flexible redox system controls B cell differentiation^31,99,100^. Compared to FoB, MZB were previously shown to use GSH through Gpx4 for protection from excessive lipid peroxidation^33^, suggesting a differential dependency on redox functions downstream of GSH synthesis. However, the impact of GSH itself, its role in B cell metabolism and B cell responses has so far been elusive. The present study has uncovered the contribution of GSH to B cell homeostasis and function. In particular, we have shown that genetic deletion of *Gclc*, and therefore loss of GSH, in B cells has important and subset-specific effects on FoB and MZB (Supplementary Fig. 7f). Our data further indicate that GSH acts as a rheostat to regulate metabolism, mitochondrial function and ETC activity in B cells.

The intracellular environment of MZB operates at a higher oxidative level compared to that in FoB. In MZB, higher levels of GSH are necessary to sustain their homeostasis and function (i.e. antibody secretion). In contrast, FoB exhibit lower intracellular ROS and maintain a metabolic setup that is distinct from that in MZB. Upon genetic deletion of *Gclc*, MZB differentiation is halted because the GSH-dependent buffering capacity is lost. Notably, FoB persist in *Gclc*-deficient mice and are characterized by increased ROS and acquire characteristics of *Gclc*-sufficient MZB. This indicates an interesting GSH-dependent threshold regulation that is important for the subset-specific functions of B cells and a differential redox-dependent regulation of metabolic activities in these cells. These metabolic states appear to be related to the divergent functions of FoB and MZB, which have been documented in numerous studies^7–14^. In particular, the differences between FoB and MZB in recirculation properties^49^, the exposure to oxygen levels, and in degree of activation upon stimulation^48^, have raised interest in their metabolic dependencies. MZB have the potential to self-renew^18^ and are activated more quickly than FoB^7,8^, properties consistent with a requirement for glycolytic reprogramming in activated B cells^101^.

The development of FoB in B cell-specific *Gclc*-deficient mice can be linked to the metabolic quiescence which characterizes wild-type FoB^38^. This basal metabolic dormancy might facilitate *Gclc*-deficient FoB to sustain the deficiency of GSH, and therefore, increased oxidative stress. Thus, when depleted of GSH, FoB experience a change in their redox buffering potential which results in a metabolic adaptation (i.e. lower mitochondrial activity but increased glycolysis) that partially mirrors the metabolic properties of wild-type MZB. In accordance with this observation, we demonstrated that resting wild-type MZB show increased glucose uptake and glycolysis compared to resting FoB. Resting wild-type FoB are instead characterized by downregulation of mTORC1 and its downstream targets^37–39^, which is indicative of metabolic quiescence. Strikingly, *Gclc* ablation increased mTORC1 activation in FoB to a level similar to that found in MZB. However, mTORC1 activation only promoted metabolic reprogramming of *Gclc*-deficient FoB but was not sufficient to ensure antibody production, in contrast to B cell cells isolated from tuberous sclerosis complex 1 (TSC1)-deficient mice^102^.

In immune cells, upregulation of metabolic pathways upon activation is necessary to generate ATP to support functions such as antibody production^101^. However, this increase in metabolic activity is also associated with elevated ROS, which are known modulators of the functions of immune cells^32,41,43,44,103^. mtROS are generated by inefficient electron transfer through the ETC^104^, and defects in the ETC have been shown to increase mtROS even further^105^. Although cells benefit from low or moderate ROS levels, high ROS impose irreversible oxidative damage and so efficient ROS removal is necessary to maintain homeostasis^21,106^ and controlled cellular activation.

Balanced mtROS are critical for the correct function of the redox-sensitive ETC proteins, which are susceptible to inactivation by these reactive molecules^107–111^. In our study, we found that activities of ETC CI and CII were reduced in GSH-deficient FoB. However, protein levels of CII and CIV were also affected, thus implying that other modifications (i.e. s-glutathionylation) or factors, such as active site availability, substrate availability, and kinetic of the reaction might play a role in the stability of these complexes. Moreover, these multi-protein complexes are continuously exposed to a GSH-depleted environment in *Gclc*-deficient FoB, which might alter protein folding or their targeting to the mitochondria. In our study, transient ROS-scavenging was not able to revert these modifications and to reinstate the functionality of these cells. This might be due to the chronic exposure to high ROS concentrations of these cells. Indeed, chronic ROS can cause protein oxidation of methionine and cysteine residues^112,113^, which in the absence of GSH becomes permanent and might decrease irreversibly CI and CII functionality. Another possible explanation why ROS-scavenging by antioxidants did not restore the functionality of *Gclc*-deficient B cells is the altered redox state caused by this treatment. Thus, antioxidants could expose these cells to reductive stress, which is also associated with cellular dysfunctionality^114,115^. We speculate that the redox state of B cells must be very precisely balanced to ensure proper functionality of these cells. Most interestingly, this is very important for B cells, while in our previous studies we could restore functionality of *Gclc-*deficient T cells by ROS-scavenging with N-acetyl cysteine and GSH^43,44^. However, we cannot rule out ROS-independent functions of GSH in B cells that contribute to our observed effects. For example GSH has been shown to be important for the regulation of cellular labile copper pools^116^. These labile copper pools are important for the assembly of iron-sulfur (FeS) clusters, which are directly linked to mitochondrial function^117,118^. Moreover, GSH-independent roles of Gclc associated with its direct product—γ-glutamylcysteine (γ-GluCys)—might influence the downstream regulation of biosynthetic pathways of amino acids (and/or their transport)^119^ in MZB and FoB.

CI activity, in particular, requires adequate levels of reduced GSH in the mitochondria for its function^120,121^. GSH-dependent mitochondrial functions are influenced by changes in levels of cytosolic GSH because GSH is synthesized in the cytosol and must be imported into the mitochondria to exert its function as antioxidant^122,123^. In addition to decreased CI, we found compromised CII activity in *Gclc*-deficient B cells. Reduced CII activity has consequences for central carbon metabolism because CII is involved in the conversion of succinate to fumarate within the TCA cycle^73^. Our metabolic analyses showed that *Gclc* deficiency led to an accumulation of succinate, indicating a block in the TCA cycle. Interestingly, we found that succinate accumulated only in *Gclc-*deficient B cells and not in *Gclc-*deficient effector T cells nor Tregs^43,44^. This result identifies an unexpected B cell-specific function of GSH in the regulation of the TCA cycle and the ETC.

Based on the results above, we suggest that GSH activity is essential to preserve ETC integrity in MZB at steady state. Because of their lower activation threshold at baseline^7^, MZB need mitochondrial GSH for their correct metabolic reprogramming and therefore *Gclc*-ablation leads to MZB loss. On the other hand, FoB rely on OXPHOS to a lower extent: *Gclc*-deficient FoB lose CI and CII functionality, but these cells increase glycolysis to replenish their ATP pool to the extent that they persist in *Gclc*^*fl/fl*^ *Mb1 Cre*^*+*^ hosts. Similarly, alterations of redox metabolism have previously been shown to have negative effects on MZB but not on FoB^103,124^. These findings support the notion that differential redox capacity exists in different B cell subsets, and suggest that steady-state MZB require greater redox buffering capacity. Our data have revealed that MZB and FoB rely on activities that differ in their dependence on GSH. Accordingly, deletion of *Gclc* in B cells leads to a specific loss of MZB content but not FoB. We have shown that GSH modulates redox functions in steady-state B cells in a subset-specific manner, adding a new element of complexity to the distinct homeostatic properties of FoB and MZB^4,48,50^. However, we cannot exclude that commitment of the FoB compartment and the reflection of wild-type MZB-like properties by FoB in B cell-specific *Gclc*-deficient hosts, might be the consequence of a physiological attempt to fill the empty MZB compartment.

Furthermore, the importance of GSH to the B cell lineage is not limited to steady-state subset-specific roles. Once FoB are activated, their demand for GSH increases to cope with accumulating ROS. Our data showed that, despite the higher mTORC1 signaling in *Gclc*-deficient FoB cells, these cells dampened antibody production and could not ensure GC formation following activation in vivo and antibody production in vitro. In a previous study, enhanced mTORC1 signaling was found to be dispensable for GC formation and serum antibody responses^102^. Thus, our data further indicate that the increased mTORC1 signaling is a consequence of GSH depletion, but it is not sufficient to sustain humoral immunity. Instead, it reflects the ability of FoB to metabolically adapt to oxidative stress.

In line, intracellular GSH levels in B cells of HIV-1-infected individuals showed a tendency to be decreased compared to uninfected donors^30,125,126^, supporting the link between GSH and the regulation of B cell activation in human infections. The effect of GSH-deficit was exacerbated upon VSV infection, resulting in the drastically reduced survival of infected *Gclc*^*fl/fl*^ *Mb1-Cre*^*+*^ mice. These results highlight the important and non-redundant role of GSH in B cell humoral immunity in vivo, but does not exclude impairment of other B cell functions such as antigen presentation or cytokine secretion.

In conclusion, we have shown that GSH is critical for B cell homeostasis and regulates metabolic pathways of MZB and FoB. GSH regulates the TCA cycle and ETC activity in a B cell-specific manner that stands in stark contrast to its functions in T cells. Our work highlights B cell-specific alterations that offer novel insights for the understanding of the role of GSH in the regulation of B cells function and defects during disease, such as those caused by viral infections.

## Methods

### Mice

Wild-type C57BL/6J (B6) were purchased from Charles River (stock number JAX 000664). *Gclc*^*fl/fl*^ mice were described previously^127^ and were crossed to *Mb1-Cre*^*+*^ expressing mice^46^ to obtain the *Gclc*^*fl/fl*^*Mb1-cre* strain. The mice were housed and bred under specific pathogen-free conditions at the Luxembourg Institute of Health (LIH) and the BTA facility of the University of Luxembourg. Female or male age-matched mice (7–12 weeks old) were used for all experiments (unless otherwise stated) and euthanasia was performed by cervical dislocation. All protocols were conducted and approved in the accordance to the LIH Animal Welfare Structure guidelines.

### Cryosections staining and microscopy

Hematoxylin and eosin (H&E) and immunofluorescence of tissues were performed on snap-frozen tissue samples in O.C.T. (Sakura 4583). For H&E staining sections were processed following standard laboratory procedures. For immunofluorescence, cryosections were dried for 2 h at RT, fixed in pure acetone for 10 min and blocked with PBS 10% FCS for 30 min at RT. After washing in PBS, primary antibodies (IgM-PE BioLegend 406507, IgD-APC BioLegend 405713, MARCO Santa Cruz sc-65353/ anti-rat PE Abcam ab97058 and Siglec-1 Biolegend 142417) diluted in PBS 10% FCS were incubated overnight at 4 °C. DAPI mounting medium (SouthernBiotech 0100-20) was used to counterstain the nucleus. For Tom20 staining (Abcam ab186734), 10^6^ FACS-sorted FoB were washed in PBS and fixed in fix/perm buffer (BD 554714) for 20 min at 4 °C. Cells were washed with perm/wash buffer (BD 554714) and fixed in fix/perm buffer. After 30 min at 4 °C, cells were washed in perm/wash buffer and resuspended with secondary Ab anti-Rabbit IgG FITC (Fisher Scientific 15303926) or PE (CST 79408 S). After the last wash, cells were resuspended in DAPI mounting medium and seeded on Cell-Tak (Fisher Scientific 10317081) coated glass slides. Tissues were visualized with ZEISS Axio Observer and cells with ZEISS LSM 880. Images were analyzed with ZEISS ZEN Blue and Fiji softwares. Nuclear and mitochondrial area were calculated using the Analyze particle function in Fiji.

For ultrastructural microscopy, 10–20 × 10^6^ FoB were fixed in complete RPMI medium + 2.5% glutaraldehyde (EMS 16220) for 1 h at RT. After spinning, supernatant was removed and pellet was resuspended in 0.1 M sodium cacodylate buffer (pH 7,4) + 2.5% glutaraldehyde and stored overnight at 4 °C. Cells were centrifuged and resuspended in 0.1 M sodium cacodylate buffer and post-fixed for 1 h with 1% osmium + 0.1 M sodium cacodylate buffer, then dehydrated and embedded in Agar 100 resin. Pelleted cells were sectioned in a RMC Boeckeler ultramicrotome using a Diatome diamond knife at a thickness setting of 60 nm. Sections were stained with 2% uranyl acetate, and lead citrate. The sections were examined using a ZEISS GEMINI 300 at 30 kV with the STEM detector. For identification of dilated intercristae space, mitochondria were counted in 25 cells per condition. Based on mitochondria from *Gclc*^*fl/fl*^ FoB (measured intercristae space 60 nm), a cutoff of >100 nm was set for identification of dilated intercristae space.

### T-dependent B cell immunization (LCMV Cl13 and VSV)

Mice were intravenously infected with 2 × 10^6^ pfu of LCMV Cl13. Blood was collected at the indicated time points for antibody detection in the serum. At the endpoint, mice were euthanized and blood, spleen, kidney, liver and lung were collected and snap-frozen until further analysis.

VSV, Indiana strain (VSV-IND, Mudd-Summers isolate), was originally obtained from D. Kolakofsky (University of Geneva, Geneva, Switzerland). Mice were infected with 10^5^ PFU of VSV. Upon appearance of clinical signs of VSV replication in the central nervous system, such as paralysis, mice were removed from the experiment.

### T-independent B cell immunization (TNP-Ficoll)

To study Ag-trapping by MZB, mice were injected i.v. with 100 µg TNP-Ficoll FITC (Biosearch Technologies F-1300F) and 30 min later tissues were analyzed with flow cytometry (splenocytes were stained with surface markers for the detection of FoB and MZB, with the addition of biotinylated anti-TNP (BD Pharmingen 554055) + Streptavidin-APC (Biolegend 405207 for TNP detection) or snap-frozen for immunofluorescence microscopy in OCT (Sakura 4583). To measure the early IgM response, the same dose of TNP-Ficoll (Biosearch Technologies F-1300-100) was administered i.p., and blood was collected one day previous injection and 3, 5, and 7 days post injection.

### Single-cell sequencing (CITE-seq): preparation, pre-processing, and analysis

For CITE-seq labelling, a total of 2 million cells/genotype were FACS sorted, counted, isolated, and spun down. The cell pellet was resuspended and incubated for 30 min on ice with 25 µL of staining mix in PBS containing 0.04% BSA, TruStain FcX Block (BioLegend 101320), and the mouse cell surface protein antibody panel containing the following oligo-conjugated anti-mouse antibodies (TotalSeq-A BioLegend) diluted 1:500: CD23 (Cat. Number 101635), IgD (Cat. Number 405745), CD1d (Cat. Number 123529), CD21/CD35 (Cat. Number 123427), IgM (Cat. Number 406535).

Sorted single-cell suspensions were resuspended at an estimated final concentration of 1000 cells/µl and loaded on a Chromium GemCode Single Cell Instrument (10x Genomics) to generate single-cell gel beads-in-emulsion (GEM). Biological replicates (*n* = 4 for each group) were multiplexed using TotalSeq-A Cell Hashing Antibodies. The scRNA/CITE-seq libraries were prepared using the GemCode Single Cell 3′ Gel Bead and Library kit, version 3 (10x Genomics 1000128) according to the manufacturer’s instructions with the addition of amplification primer (3 nM, 5′CCTTGGCACCCGAGAATT*C*C) during cDNA amplification to enrich the TotalSeq-A cell surface protein oligos. Sequencing libraries were loaded on an Illumina HiSeq4000 flow cell at VIB Nucleomics core with sequencing settings according to the recommendations of 10x Genomics, pooled in a 85:15 ratio for the gene expression and antibody-derived libraries, respectively. The Cell Ranger pipeline (10x Genomics version 3.1.0) was used to perform sample demultiplexing and to generate FASTQ files for read 1, read 2, and the i7 sample index for the gene expression and cell surface protein libraries. Read 2 of the gene expression libraries was mapped to the reference genome (mouse mm10, v3.0.0) using STAR. For cellular identification and clustering of the gene-barcode matrix was passed to the R (version 4.04) package Seurat (v. 4.0)^128^ for all downstream analyses. Analysis was conducted on cells that expressed a minimum of 800 genes, of which less than 15% was of mitochondrial origin. Count data were derived through the SCTransform function. To identify FoB and MZB, Antibody-derived signals (ADT) were used in combination with the SCINA algorithm^51^ with the following signatures: CD23-ADT, IgD-ADT for FoB; CD1d-ADT, CD21-CD35-ADT, IgM-ADT for MZB. For principal component analysis (PCA), counts of each cell group/mouse for each genotype where extracted and analyzed using DESeq2^129^. Cluster-based marker identification and differential expression were performed using Seurat’s FindMarkers/DESeq2 with logfc.threshold = 0.2 comparing specific groups (i.e. MZB vs. FoB for *Gclc*^*fl/fl*^, or FoB *Gclc*^*fl/fl*^ *Mb1 Cre*^*+*^ vs. FoB *Gclc*^*fl/fl*^). Visualization of differentially expressed genes was done with EnhancedVolcano^130^. For gene ontology (GO) analysis, the Seurat’s FindMarkers output for the specified groups was used to plot gene ontology terms with the barcodeplot functions from limma^131^. For GSEA analyses, the fgsea package was used^132^.

scRNA-seq data were analyzed as above and the gene expression matrix of 156 cells/group (MZB and FoB from *Gclc*^*fl/fl*^ mice) or 1700 FoB/genotype (i.e. *Gclc*^*fl/fl*^ or *Gclc*^*fl/fl*^ *Mb1 Cre*^*+*^ mice) was used as input for the Compass algorithm^70,71^. Downstream analysis was conducted with the compassR package and differential metabolic states were determined with a Wilcoxon Rank Sum Test on the Compass reactions.

### Flow cytometry and sorting

For surface staining cells were incubated at 4–8 °C, in the dark with fluorochrome-conjugated CD19, CD23, CD21-CD35 or CD35, IgD, IgM, CD1d, CD24, CD93, CD95, and GL-7 diluted 1:200 (refer to Supplementary Data 4 for details). The antibody mix was washed from the cells after 20–30 min. Samples from in vivo LCMV experiments were fixed for 10 min at RT in a volume of at least 100 μL 2% formaldehyde (FA) after staining. All antibodies dilutions are made from stock solutions prepared as per the manufacturer’s instructions, where applicable.

For intracellular staining of phospho(p)-mTOR (Fisher Scientific 15549836) and p-S6 (eBioscience 15528216), cells were first stained for extracellular markers, fixed in 2% FA at RT, and stained for p-mTOR and p-S6 diluted 1:200 in saponin 5 µg/mL. p-eIF4E staining was done using mouse anti-elF4E (pS209) (BD 560229) diluted 1:100 and cells were fixed with BD Cytofix/Perm and intracellular staining was done in BD Perm (BD 554714). For intracellular staining of hexokinase-1, cells were stained for extracellular markers, fixed in 4% FA at RT, and stained with Anti-Hexokinase 1 (Abcam ab184818) diluted 1:100 in PBS 0.1% Tween-20 at RT. For viability, 7-aminoactinomycin D (7AAD) (Thermo fisher A1310) or Zombie NIR (BioLegend 423106) diluted 1:3000 was stained concurrently with antibodies in all flow cytometry assays. For glucose uptake and GLUT-1 surface detection splenocytes were washed with glucose-free RPMI medium (Lonza BE12-752F). Pellet was resuspended in glucose-free RPMI medium supplemented with 2-NBDG (Thermo Fisher N13195) at 50 µM and incubated for 30 min at 37 °C. Anti-GLUT1 (Abcam ab195359) diluted 1:200 was used and expression was measured on FoB gated on total splenocytes as described above. For detection of DRP1, cells were washed and fixed in 4% FA at RT after surface staining. Then, cells were washed and stained in Triton 0.1% containing DRP1 antibody (Abcam ab184247) diluted 1:100. After 30 min at RT, cells were washed and incubated with secondary Ab anti-Rabbit IgG FITC (Fisher Scientific 15303926).

Sdha detection was performed on FoB from total spleen with the MitoBiogenesis Flow Cytometry Kit (Abcam ab168540) following the manufacturer’s instructions. Lipid peroxidation was measured with using Bodipy 581/591 C11 (Fisher Scientific D3861) at 1 µM following the manufacturer’s protocol. For quantitation of mitochondrial potential and mass of FoB and MZB, splenocytes were stained with 100 nM MitoTracker Deep Red FM and 10 nM MitoTracker Green FM (Fisher Scientific 15754272 and 15784272) as follows. Splenocytes were washed with warm RPMI 1640, stained at 37 °C for 30 min with Abs diluted in RPMI 1640, and washed with PBS. To detect intracellular ROS, cells were incubated with dichlorofluorescein diacetate (Carboxy-H2DCFDA; Thermo Fisher 11500146) diluted 1:5000 or MitoSOX Red (Thermo Fisher 11579096) diluted 1:2500 as follows. Splenocytes were washed with warm RPMI 1640, stained for surface markers detection at 37 °C for 30 min in RPMI 1640, and washed with PBS. Detection of cellular thiols was done using monobromobimane (MBB, ThermoFisher M1378) added 10 min before acquisition at 50 µM. Mitochondrial thiols were detected by measuring the MFI of MBB in CD19^−^ Tom20^+^ (Abcam ab186734) isolated mitochondria from the enriched FoB and MZB fractions. NADH levels were measured in FoB from splenocytes, and were measured as mean autofluorescence intensity upon excitation with UV laser and recorded with a band pass filter BP465/30^133,134^. ER and Golgi apparatus were stained using ER-Tracker Red (Fisher Scientific 11584746) diluted 1:2000 and anti-Giantin (BioLegend 908701)^135^ diluted 1:500. Briefly, after surface staining, cells were fixed in FA and permeabilized with Triton 0.1%. ER and Golgi stain were performed in Triton 0.1%, 20 min, RT. Annexin V^+^ cells were detected upon 5 h treatment with 2-Deoxy-D-glucose. Briefly, FoB were washed and stained with Annexin binding buffer (Invitrogen BMS500BB). Cells were stained with CD19, 7-AAD, and anti-Annexin V-PE diluted 1:200 (BioLegend, 640908) for 20 min, RT.

Cell counts (cellularity) was determined as follows for each individual tube: 10^4^ unlabeled beads (BD Calibrite 3, 340486) were added prior to acquisition. Beads were distinguished from lymphocytes through FSC-A/SSC-A profile. Beads count and the cells of interest (i.e. total live CD19^+^ cells) were retrieved with FlowJo v10.6.1. Cell counts of the population of interest was normalized by the beads counts acquired. The following calculation was used:

$${{{{{\rm{Cellularity}}}}}}\,\left({{{{{\rm{counts}}}}}}\right)=\frac{{{{{{\rm{Acquired}}}}}}\,{{{{{\rm{number}}}}}}\,{{{{{\rm{of}}}}}}\,{{{{{\rm{cells}}}}}}\; {{{{{\rm{of}}}}}}\; {{{{{\rm{interest}}}}}}}{{{{{{\rm{acquired}}}}}}\; {{{{{\rm{total}}}}}}\; {{{{{\rm{live}}}}}}\; {{{{{\rm{lymphocytes}}}}}}}\times\frac{{10}^{4}}{{{{{{\rm{acquired}}}}}}\; {{{{{\rm{number}}}}}}\; {{{{{\rm{of}}}}}}\; {{{{{\rm{beads}}}}}}}$$

FoB cell sorting for confocal microscopy and TEM was performed by MACS pre-enrichment with B cell isolation kit (Miltenyi Biotech 130-090-862). Untouched B cells were then stained with CD19, CD21/CD35, and CD23. Live CD23^high^CD21/35^low^ FoB cells were FACS-sorted using Aria IIu (BD). For CITE-seq, 10^4^ B cells were identified as CD11b^−^TCR-beta^−^CD19^+^B220^+^ and FACS-sorted using Aria II (BD). Flow cytometry was performed using a BD Fortessa instrument (BD) or NovoCyte Quanteon and data were analyzed using FlowJo v10.6.1 software (Tree Star).

### Cell culture and assays

Total B cells and FoB/MZB were magnetically enriched from mouse spleen using a B cell or MZ and FO B Cell Isolation Kit, respectively (Miltenyi Biotec 130-090-862 and 130-100-366) as per manufacturer’s protocol. Mitochondria were isolated (Miltenyi Biotec 130-096-946) from 10 × 10^6^ pooled cells from 2 to 3 mice following the manufacturer’s protocol. Most of the experiments were performed with MACS-sorted FoB cells, unless clearly specified. Cells were seeded in complete medium consisting of RPMI-1640 supplemented with 10% FCS (Sigma), 1% Penicillin/Streptomycin (GIBCO), 1% L-Glutamine (Sigma), and 55 mM β-mercaptoethanol (GIBCO). Cells were seeded in 96-well plates at 2 × 10^5^ cells/well unless otherwise indicated. FoB were activated with 5 µg/mL anti-IgM (Jackson Immunoresearch 715-006-020), 50 ng/mL CD40 ligand (Bio-techne 8230-CL-050) and 10 ng/mL IL-4 (Miltenyi Biotec 130-097-757). Reduced GSH was purchased from Sigma (G4251). MZB were stimulated with 1ug/mL of LPS (Sigma L2630). To inhibit glycolysis, 2-Deoxy-D-glucose (2-DG, Sigma D8375) or galactose (D-(+)-Galactose, Sigma G0750) was used at the indicated concentration. The GSH/GSSG content and ATP levels of 0.5–1 × 10^5^ cells/well were measured by a luminescent-based assay (Promega V6612 for GSH/GSSG and Promega G7571 or Sigma MAK135 for ATP) following the manufacturer’s protocol. Complex I activity was measured with the Complex I Enzyme Activity Kit (Abcam ab109721). To inhibit mitochondrial metabolism and GSH synthesis, the following chemicals were purchased from Sigma: metformin, rotenone, antimycin A, oligomycin A and BSO.

To measure PIP_3_, FoB were stimulated with 5 µg/mL anti-IgM (Jackson Immunoresearch 715-006-020) for the indicated time points, then fixed in 2% FA and permeabilized with 0.1% saponin. Biotinylated Anti-PtdIns(3,4,5)P3 IgG (echelon biosciences Z-B345b) and Invitrogen eBioscience Streptavidin-PE (Fisher scientific 11500607) were used for detection.

### ELISAs

ELISA assays were performed in 96-well NUNC plates (Fisher Scientific 11371605) unless otherwise indicated. Readings were recorded with SpectraMax ELISA plate reader instrument (Molecular Devices). Washes were done with PBS Tween 0.05% (PBS-T).

Detection of CXCL13 (biotechne MCX130) and S1P (tebu-bio K-1900-1ea) were measured in the serum following the manufacturer’s recommendation. For total Ig detection from cell supernatants, plates were coated at 4 °C overnight with goat Anti-Mouse Ig (Southern Biotech 1010-01) at 1 µg/mL. After tipping off the supernatant, plates were washed, blotted on paper and blocked with PBS 1% BSA for 30 min at RT. After tipping off the supernatant, diluted supernatants were incubated for 2 hr at RT. After washing, 50 μL/well of HRP-conjugated detection antibody anti-Ig-HRP (Cytiva NA9310-1ML) was added for 1 h at RT. Plates were developed by adding 50 µL of RT TMB solution (ThermoFisher 12750000). Reaction was stopped by adding 2N H_2_SO_4_ and absorbance was recorded at 450 nm.

For TNP-specific antibody detection, plates were coated at 4 °C overnight with NP_(7)_-BSA (Biosearch Technologies N-5050L) at 1 µg/mL. After tipping off the supernatant, plates were washed, blotted on paper, and blocked with PBS 1% BSA for 30 min at RT. After tipping off the supernatant, diluted serum samples were incubated for 2 h at RT. After washing, 50 μL/well of HRP-conjugated detection antibody (anti-IgM-HRP Sigma A8786) was added for 1 h at RT. Plates were developed by adding 50 µL of RT TMB solution (ThermoFisher 12750000). Reaction was stopped by adding 2N H_2_SO_4_ and absorbance was recorded at 450 nm.

Analysis of IgM and IgG anti-LCMV-Gp specific antibodies was performed as previously described^93^. Briefly, plates were coated overnight with anti-human Fc (Jackson ImmunoResearch Laboratories 109-001-008), blocked for 2 h with PBS 2% BSA at RT, and incubated overnight at 4 °C with recombinant Gp1-Fc protein derived from HEK293 cells transfected with LCMV glycoprotein vector. Diluted sera of LCMV Cl13 infected mice were serially diluted and incubated for 90 min at RT. Plates were washed with PBS-T and anti-mouse IgM-HRP or anti-mouse IgG-HRP (Sigma A8786 and A3673) was used for detection. Plates were washed and a green color reaction was produced with 2,2′azino-bis(3-ethylbenzothiazoline-6-sulfonate) (ABST, Sigma 10102946001) diluted in 0.1 M NaH_2_PO_4_ (pH = 4). Plates were read at 405 nm and titers were defined as the log_3_ serum dilution two-fold above background.

### RNA, DNA isolation and PCR

RNA was isolated using NucleoSpin RNA Kit (Macherey-Nagel 740955250). RT-qPCR was carried out using Luna Universal One-Step RT-qPCR Kit (Bioké E3005E) with the following primers: *Gclc* forward (F) GGCTCTCTGCACCATCACTT and reverse (R) GTTAGAGTACCGAAGCGGGG; *Tbp* F GAAGAACAATCCAGACTAGCAGCA and R CCTTATAGGGAACTTCACATCACAG; *Cox1* F GCCCCAGATATAGCATTCCC and R GTTCATCCTGTTCCTGCTCC; *18S* F TAGAGGGACAAGTGGCGTTC and R CGCTGAGCCAGTCAGTGT.

Reactions were run on a CFX384 instrument (Bio-Rad). Data were normalized to *Tbp* and analyzed using the ΔΔCt method as previously described^136^. DNA isolation from MACS-sorted FoB and MZB was performed with NucleoSpin 96 Tissue kit (Macherey-Nagel 740454.4). RT-qPCR was carried out using Fast SYBR Green Master Mix (Thermo Fisher 4385610) and the mitochondrial/nuclear DNA ratio was derived as described previously^137^.

### Immunoblot analysis

For detection of Gclc (Santa Cruz Biotechnology sc-390811), Pdha-1 (Abcam ab168379), DRP1 (Abcam ab184247), eIF4E (Abcam ab33766), p-eIF4E (Abcam ab76256), 4E-BP1 (Cell signaling 9452), p-4E-BP1 (Cell signaling 2855), DRP1 (Abcam ab184247), mTOR (Cell Signaling 2972), p-mTOR (Ser2448) (Cell Signaling 2971), p70 S6 (Cell Signaling 9202), p-p70 S6 (Thr421/Ser424) (Cell Signaling 9204), 1–5 × 10^6^ cells were lysed with lysis buffer (CST 9803S) and protein/phosphatases inhibitors as per manufacturer’s protocol and blotted as described^44^. Briefly, cells were washed and lysed on ice for 30 min and spun. Supernatants were assayed for total protein concentration with Bradford assay (BioRad 5000006) and 50–100 µg of protein was mixed with loading buffer and loaded in a 12 or 16% gradient gel (Thermo 1Fisher XP00162). Proteins were transferred onto a NC or PVDF membrane (Fisher Scientific) and blocked with 5% milk for 1 h. OXPHOS complexes were revealed with total OXPHOS Rodent WB Antibody Cocktail (Abcam ab110413). Lysis was carried out with the addition of lauryl maltoside at 1.5%.

### Isotopic labelling and mass spectrometry

FoB cells were incubated for 5 h in SILAC RPMI containing [U-^13^C_6_]-glucose (11 mmol/L; Cambridge Isotope Laboratories). Extraction of intracellular metabolites, GC-MS measurement, MID calculations, determinations of fractional carbon contributions, and subtractions of natural isotope abundance were performed as described^44^. Total glucose and lactate concentrations from medium were determined using a YSI 2950D. Intracellular metabolites (GSH, glucose, glucose-6P, pyruvate, succinate) were measured by liquid chromatography (Vanquish HPLC Thermo Scientific) coupled to high resolution mass spectrometry (QExactive HF Thermo Scientific). Briefly, extraction of 3–30 × 10^5^ cells was done in a 2:2:1 mixture of acetonitrile:MeOH:H_2_O + 0.5% formic acid on ice. Following neutralization with (NH)_4_HCO_3_, samples were frozen at −20 °C for 20 min, spun and supernatants measured directly.

### Seahorse flux analysis

Assays were performed using an XFe96 Extracellular Flux Analyzer (Agilent). FoB or MZB cells were seeded in XF Seahorse RPMI medium at 10^6^/well on Seahorse XFe96 culture plates pre-coated with Cell-Tak. Oxygen consumption rate (OCR) of intact cells were determined using the XF Cell Mitochondrial Stress Test according to the manufacturer’s protocol. Briefly, three basal OCR measurements were taken, followed by sequential injections of 1 µM oligomycin A, 3 µM FCCP, and 1 µM antimycin A, taking three measurements following each treatment. GlycoATP and mitoATP were derived from cells sequentially treated with oligomycin A and Rot/Antimycin A. Activity measurements for each respiratory chain complex in permeabilized cells were performed according to previous research^85,138^. Briefly, after enrichment, FoB were washed and resuspended in mannitol and sucrose-BSA (MAS-B) buffer pH 7.2 (70 mM sucrose, 220 mM Mannitol, 10 mM KH_2_PO_4_, 5 mM MgCl_2_, 2 mM Hepes, 1 mM EGTA, and 0.4% fatty acid-free BSA), and flux measurements were started. After three basal measurements, cells were permeabilized by injection of saponin (1 µg/mL, Sigma S4521), together with 1 mM ADP (Sigma 01905) and the following respiratory complex substrates: CI, pyruvate/malate (5 mM/2.5 mM); CII, succinate/rotenone (5 mM/1 µM); CIII, duroquinol (0.5 mM); CIV, N,N,N,N-tetramethyl-p-phenylenediamine (TMPD)/ascorbate (0.5 mM/2 mM). These were followed by injections with oligomycin A (1 µM) and respective complex inhibitors (CI, 1 µM rotenone; CII and CIII, 20 µM antimycin A; CIV, 20 mM potassium azide).

### Statistical analyses

Data are graphed as the mean ± SD (refer to figure legends for detailed information) and *P* value were determined by unpaired Student’s t-test (two-tailed), one-way or 2 way ANOVA using Prism (GraphPad version 9.0.1). Significance is indicated with asterisks * and a minimum *P* value of 0.05 was considered statistically significant (refer to figure legends for details).

### Reporting summary

Further information on research design is available in the Nature Research Reporting Summary linked to this article.

## Supplementary information

Supplemental Information

Description of additional Supplementary File

Supplementary Dataset 1

Supplementary Dataset 2

Supplementary Dataset 3

Supplementary Dataset 4

Reporting Summary

## Data Availability

The data sets generated during and/or analyzed during the current study are available upon reasonable request. The CITE-seq data from control and *Gclc*-deficient B cells have been deposited in GEO under the accession code GSE194419. Where applicable, source data are provided as a Source Data file for each figure. Source data are provided with this paper.

## Source data

Source Data
