# Supplementary material for: Glutathione-dependent redox balance characterizes the distinct metabolic properties of follicular and marginal zone B cells
Source: Nat Commun. 2022 Apr 4;13:1789. doi: 10.1038/s41467-022-29426-x (PMC8980022; doi:10.1038/s41467-022-29426-x)
Supplement: Supplementary file 1 — Supplemental Information [file 41467_2022_29426_MOESM1_ESM.pdf]

# Supplementary Figures

## Glutathione-dependent redox balance characterizes the distinct metabolic properties of follicular and marginal zone B cells

D. G. Franchina *et al*.

Supplementary Figure 1. Exemplary gating of the main strategies for flow cytometry and cell sorting.

| MFI | Fig. or Supp. |
|---|---|
| DCFDA | 1c; 2f; S2a |
| MitoSOX | 1d; 2g; S2b |
| MBB | 1e; S3g; S6g |
| p-mTOR | 3b; 3e |
| p-S6 | 3c; 3f |
| PIP3 | 3g |
| 2-NBDG | 4b; 4f |
| Glut-1 | 4c; 4g |
| MT-Red | 5k |
| MT-Green | 5k |
| Sdha | 6m |
| BODIPY C11 | S2d |
| ICAM-1 | S3a |
| VCAM-1 | S3a |
| ER-Tracker | S5b, g |
| Giantin | S5b, g |
| p-eIF4E | S5c, h |
| Puromycin | S5f, k |
| Hexokinase-1 | S6b |
| TNP | S7a |

a

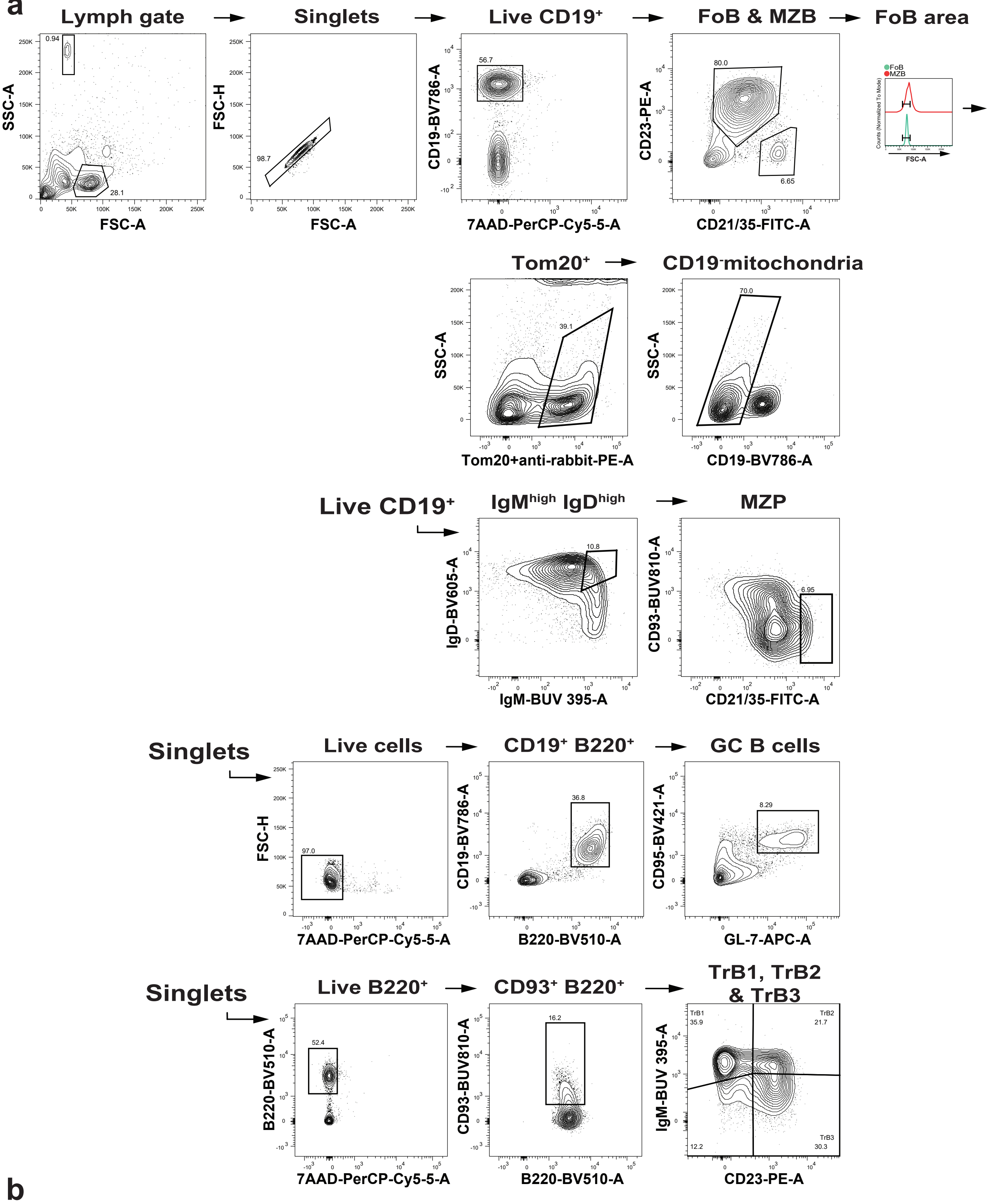


b

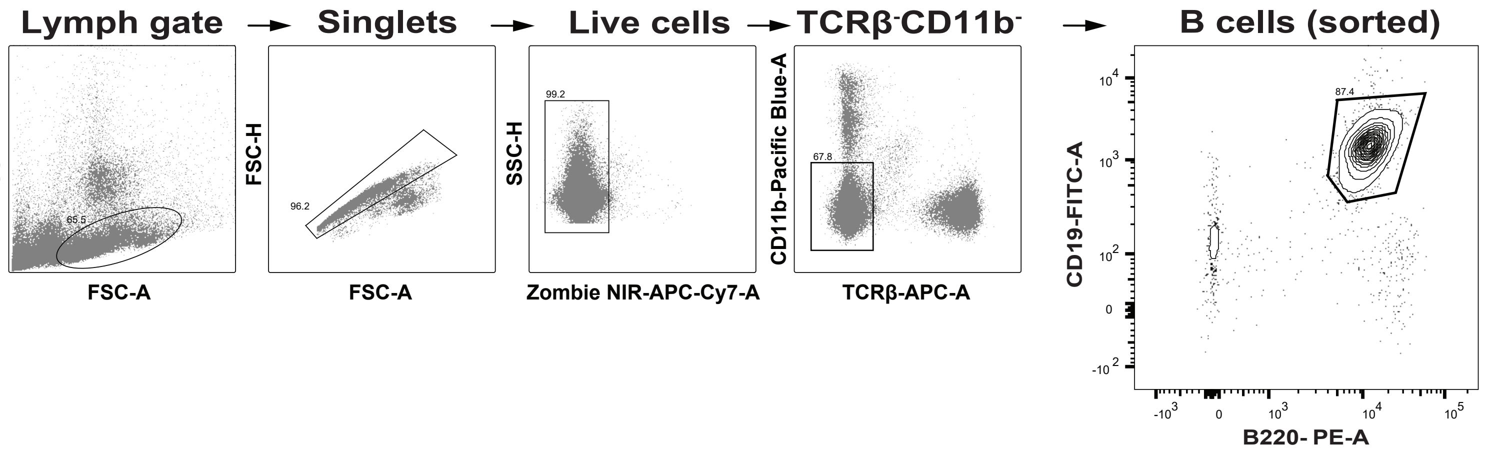

**Supplementary Figure 1. Exemplary gating of the main strategies for flow cytometry and sorting.**

**(a)**. General and representative flow cytometry gating strategy for gating of FoB, MZB, Tom20$^+$ mitochondria, MZP, GC B cells, transitional B cells (TrB1, TrB2 and TrB3). For mean fluorescence intensity (MFI) measurements, signal from the MZB gate was size-corrected based on the FSC-A signal from FoB as shown **(b)**. Flow cytometry back-gating strategy for sorting splenic B cells for scRNA-sequencing. Numbers in contour plots represent percentages of the cells gated.

Supplementary Figure 2. *Gclc* deletion led to ROS increase in splenic B cells and loss of MZB, which is *Cre*-independent.

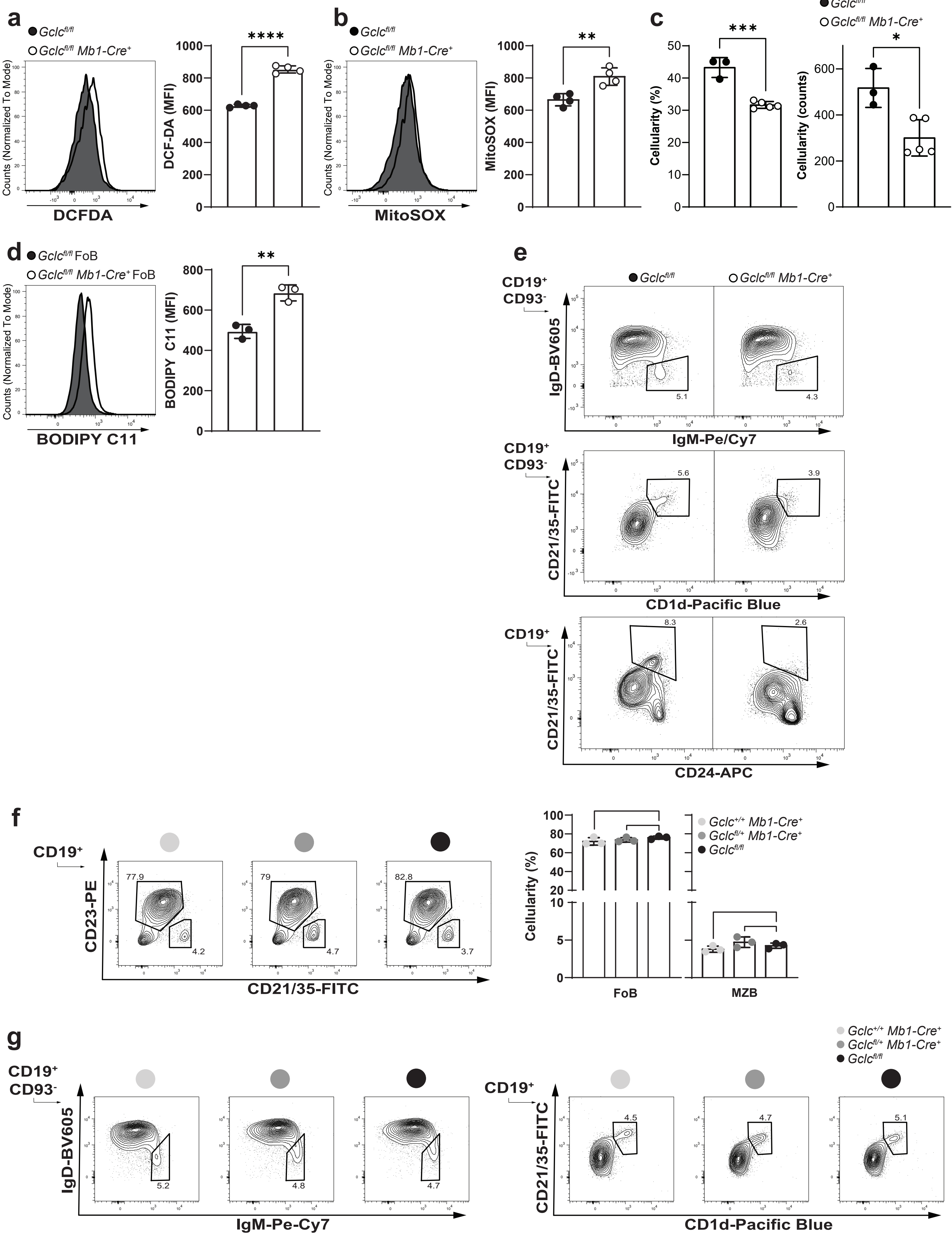

**Supplementary Figure 2. *Gclc* deletion led to ROS increase in splenic B cells and loss of MZB, which is *Cre*-independent.**

**(a-b)** Representative histogram (left) and quantitation (right) of DCFDA staining for intracellular ROS and MitoSOX for mitochondrial ROS detection in total splenic $CD19^{+}$ B cells from $Gclc^{fl/fl}$ and $Gclc^{fl/fl}$ $Mb1\text{-}Cre^{+}$ mice (gated as in Supplementary Figure 1a) (n = 4 animals examined over 3 independent experiments). **(c)** Percentages (left) and absolute numbers (right) of viable splenic $CD19^{+}$ cells from $Gclc^{fl/fl}$ and $Gclc^{fl/fl}$ $Mb1\text{-}Cre^{+}$ mice (gated as in Supplementary Figure1a) (n = 3-5 animals examined over 3 independent experiments). **(d)** Representative histogram (left) and quantitation (right) of BODIPY C11 staining for lipid ROS detection in splenic FoB cells from $Gclc^{fl/fl}$ and $Gclc^{fl/fl}$ $Mb1\text{-}Cre^{+}$ mice (gated as in Supplementary Figure 1a) (n = 3 animals examined over 2 independent experiments). **(e)** Representative contour plots of the expression of the indicated MZB markers gated as indicated from total splenocytes of $Gclc^{fl/fl}$ and $Gclc^{fl/fl}$ $Mb1\text{-}Cre^{+}$ mice. **(f)** Representative FACS contour plot (left) and quantitation (right) of splenic FoB and MZB from $Gclc^{+/+}$ $Mb1\text{-}Cre^{+}$, $Gclc^{fl/+}$ $Mb1\text{-}Cre^{+}$ and $Gclc^{fl/fl}$ $Mb1\text{-}Cre^{-}$ mice (gated as in Supplementary Figure 1a) (n = 3 animals examined over 2 independent experiments). **(g)** Representative contour plots of the indicated MZB markers expressed by total splenocytes of $Gclc^{+/+}$ $Mb1\text{-}Cre^{+}$, $Gclc^{fl/+}$ $Mb1\text{-}Cre^{+}$ and $Gclc^{fl/fl}$ mice. In contour plots numbers represent percentages of the cells gated. Data are the mean ± SD. Each dot represents one mouse. Significance (P) was calculated with unpaired t-test, except for (f) (one-way ANOVA). *: P ≤ 0.5; **: P ≤ 0.01; ***: P ≤ 0.001; ****: P ≤ 0.0001.

Supplementary Figure 3. Loss of MZB content in *Gclc*-deficient mice is due to defective maturation of B cell precursors.

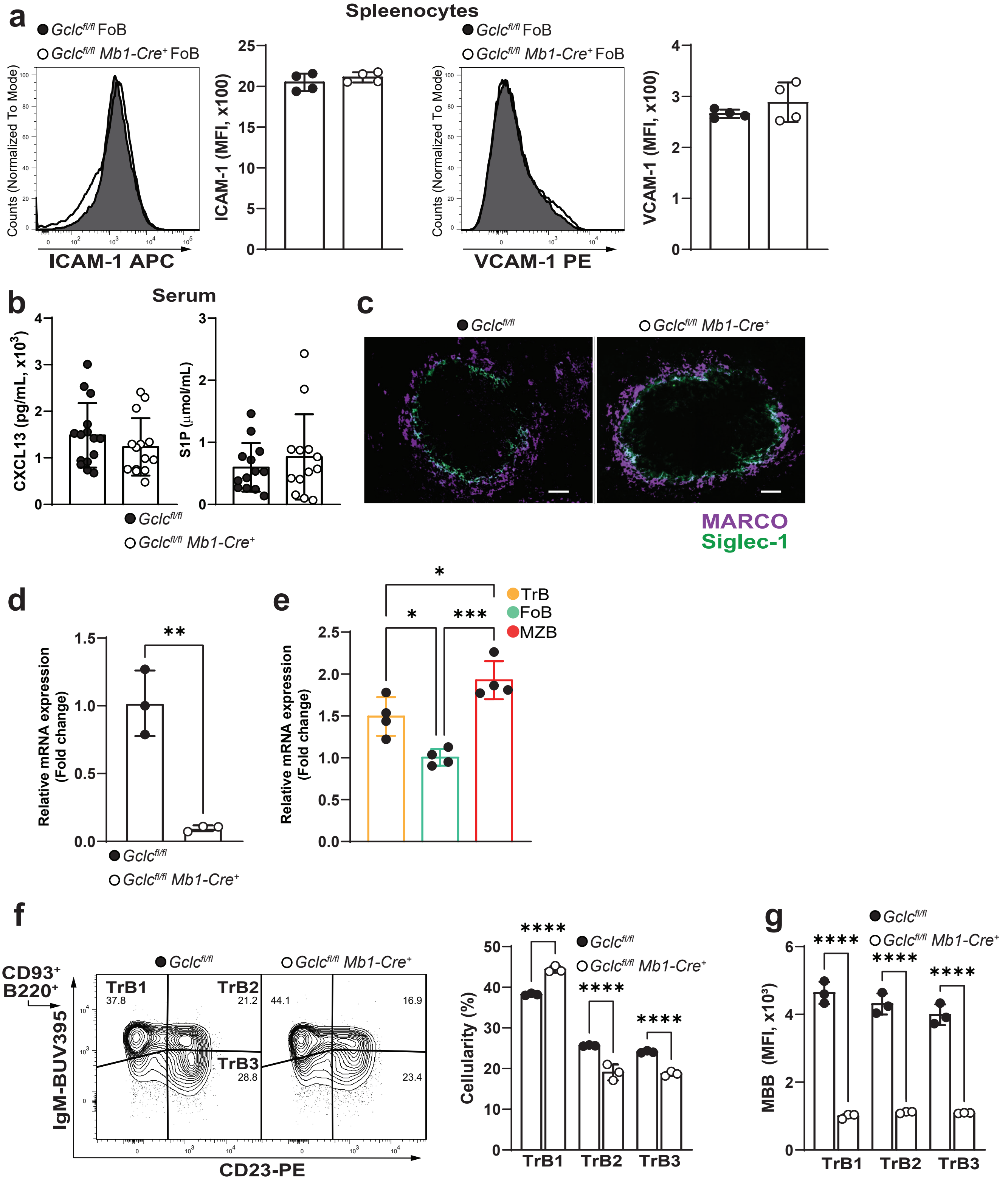

**Supplementary Figure 3. Loss of MZB content in *Gclc*-deficient mice is due to defective maturation of B cell precursors.**

**(a)** Representative histogram (left) and MFI statistic of ICAM-1 and VCAM-1 staining in total live splenocytes from *Gclc*$^{fl/fl}$ and *Gclc*$^{fl/fl}$ *Mb1-Cre*$^{+}$ mice (n = 4 animals examined over 3 independent experiments). **(b)** ELISA quantitation of CXCL13 and S1P from serum of *Gclc*$^{fl/fl}$ and *Gclc*$^{fl/fl}$ *Mb1-Cre*$^{+}$ mice (n = 3-5 animals examined over 4 independent experiments; data are pooled from 3 experiments). **(c)** Representative immunofluorescence staining to detect MARCO$^{+}$ metallophilic macrophages (purple) and Siglec-1$^{+}$ marginal zone macrophages (green) in a single spleen follicle resected from one *Gclc*$^{fl/fl}$ and one *Gclc*$^{fl/fl}$ *Mb1-Cre*$^{+}$ mouse. Scale bars: 50 µm. **(d-e)** RT-qPCR of *Gclc* mRNA expression in resting total sorted CD93$^{+}$ TrB from spleen of splenocytes from *Gclc*$^{fl/fl}$ and *Gclc*$^{fl/fl}$ *Mb1-Cre*$^{+}$ mice (d) and from resting FoB (green), MZB (red) and TrB (orange) isolated from spleen of B6 mice (e) (n = 3 animals examined over 2 independent experiments). **(f)** Representative contour plot (left) and percentages (middle) statistic of Trb1-3 from *Gclc*$^{fl/fl}$ and *Gclc*$^{fl/fl}$ *Mb1-Cre*$^{+}$ mice (gated as in Supplementary Figure 1a) (n = 3 animals examined over 3 independent experiments). **(g)** Mean fluorescence intensity (MFI) of MBB staining for detection of intracellular thiols from the cells gated in (f) from *Gclc*$^{fl/fl}$ and *Gclc*$^{fl/fl}$ *Mb1-Cre*$^{+}$ mice (n = 3 animals examined over 2 independent experiments). In contour plots numbers represent percentages of the cells gated. Data are the mean ± SD. Each dot represents one mouse. Significance (P) was calculated with unpaired t-test except for (e) (one-way ANOVA) and (f)-(g) (2way ANOVA). *: $P \leq 0.5$; **: $P \leq 0.01$; ***: $P \leq 0.001$; ****: $P \leq 0.0001$.

Supplementary Figure 4. CITE-seq antibody-derived tag (ADT) signals identifies FoB and MZB from total splenic B cells.

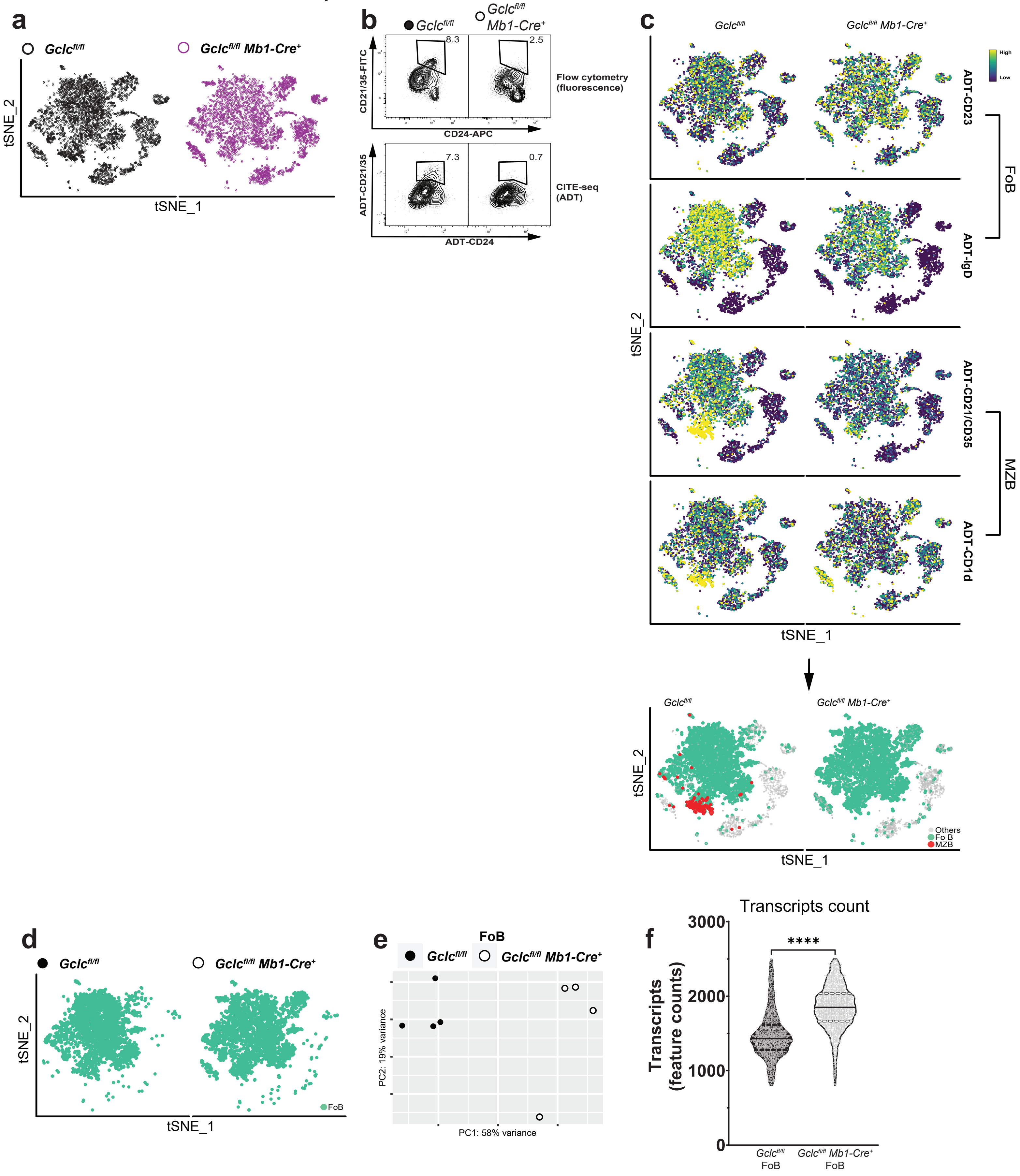

**Supplementary Figure 4. CITE-seq antibody-derived tag (ADT) signals identifies FoB and MZB from total splenic B cells.**

**(a)** tSNE distribution of single cell transcriptomes of FACS sorted splenic B cells from of $Gclc^{fl/fl}$ and $Gclc^{fl/fl}$ *Mb1-Cre*$^{+}$ mice (gated as in Supplementary Figure 1b). **(b)** Comparison of FACS (fluorescence) and CITE-seq signal (ADT) for the detection of MZB in $Gclc^{fl/fl}$ and $Gclc^{fl/fl}$ *Mb1-Cre*$^{+}$ mice. **(c)** Top: tSNE plots comparing ADT signals for FoB (top) and MZB (bottom) markers among splenic B cells from $Gclc^{fl/fl}$ and $Gclc^{fl/fl}$ *Mb1-Cre*$^{+}$ mice. Bottom: SCINA assignments of FoB (green) and MZB (red) from $Gclc^{fl/fl}$ and $Gclc^{fl/fl}$ *Mb1-Cre*$^{+}$ mice. **(d)** SCINA assignments of FoB from $Gclc^{fl/fl}$ and $Gclc^{fl/fl}$ *Mb1-Cre*$^{+}$ mice used for downstream analyses. **(e)** PCA plot showing distinct patterns of $Gclc^{fl/fl}$ and $Gclc^{fl/fl}$ *Mb1-Cre*$^{+}$ FoB transcriptomes (n = 4 mice/genotype). **(f)** Number of detected gene transcripts per cell in $Gclc^{fl/fl}$ and $Gclc^{fl/fl}$ *Mb1-Cre*$^{+}$ FoB. In contour plots numbers represent percentages of the cells gated. Data are pooled from 4 mice/genotype. Solid line, median; dashed lines, 1$^{st}$ and 3$^{rd}$ quartiles. Data are the mean ± SD. Significance (P) was calculated with unpaired t-test. ****: $P \leq 0.0001$.

Supplementary Figure 5. Protein synthesis is enhanced in *Gclc*-deficient FoB, similarly to *Gclc*-sufficient MZB.

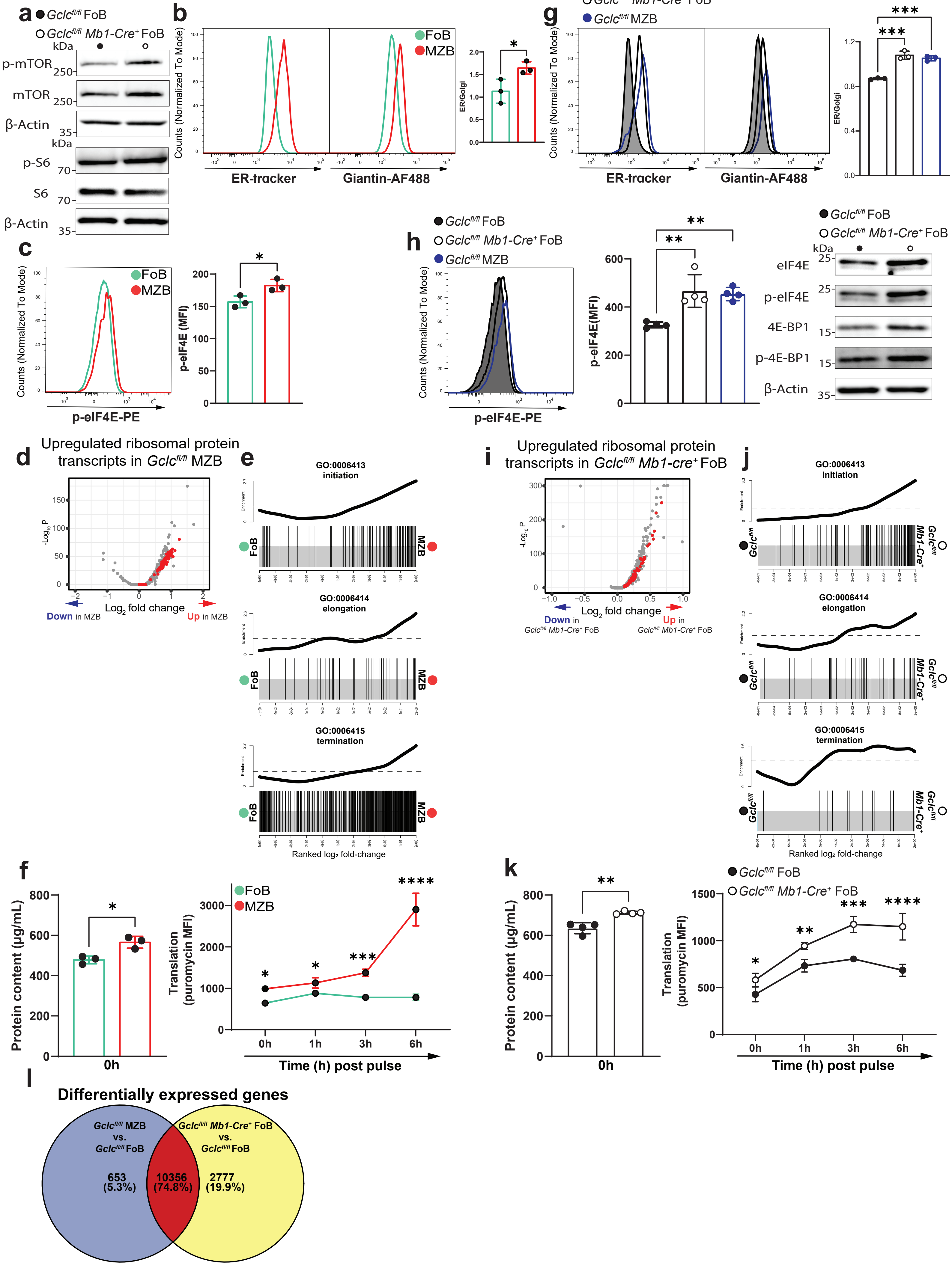

**Supplementary Figure 5. Protein synthesis is enhanced in *Gclc*-deficient FoB, similarly to *Gclc*-sufficient MZB.**

**(a)** Representative blot of p-mTOR, total mTOR and p-S6, total S6 proteins from total cell lysis of *Gclc*$^{fl/fl}$ and *Gclc*$^{fl/fl}$ *Mb1-Cre*$^{+}$ FoB is shown. **(b-c)** Representative histogram (left) and quantitation (right) of immunostaining to detect ER-tracker and Giantin (b) and p-eIF4E (c) in B6 FoB and MZB (gated as in Supplementary Figure 1a) (n = 3 animals examined over 3 independent experiments). **(d)** Volcano plot of genes differentially expressed in *Gclc*$^{fl/fl}$ MZB and FoB determined as in Fig. 1i. Red dots indicate upregulated ribosomal-related transcripts. Data represent up- and downregulation of MZB vs. FoB transcripts among total transcripts pooled from 4 *Gclc*$^{fl/fl}$ mice. **(e)** Barcode plots showing transcripts from the GO:0006413, GO:0006414 and GO:0006415 gene lists that were enriched in *Gclc*$^{fl/fl}$ MZB vs. FoB. **(f)** Protein quantitation from resting B6 0.5x10$^{6}$ cells FoB and MZB (left) and protein translation at the indicated times measured as puromycin MFI after puromycin pulse-chase of B6 FoB and MZB upon stimulation with anti-IgM, CD40 ligand and IL-4 or LPS (n = 3 animals examined over 3 independent experiments). (**g-k**) Same analyses as shown in (b-d) but comparing *Gclc*$^{fl/fl}$ versus *Gclc*$^{fl/fl}$ *Mb1-Cre*$^{+}$ FoB. (g) and (h) (n = 3 animals examined over 3 independent experiments) show comparison of *Gclc*$^{fl/fl}$ and *Gclc*$^{fl/fl}$ *Mb1-Cre*$^{+}$ FoB, including *Gclc*$^{fl/fl}$ MZB as control (gated as in Supplementary Figure 1a). In (h) a representative blot of eIF4E, p- eIF4E, 4E-BP1 and p-4E-BP1 proteins from total cell lysis of *Gclc*$^{fl/fl}$ and *Gclc*$^{fl/fl}$ *Mb1-Cre*$^{+}$ FoB is shown. (**j**) Venn diagrams show the percentages and numbers of transcripts overlap (red) of total differentially expressed (DE) genes between the DE analysis *Gclc*$^{fl/fl}$ MZB vs. FoB (blue) and *Gclc*$^{fl/fl}$ *Mb1-Cre*$^{+}$ FoB vs *Gclc*$^{fl/fl}$ FoB (yellow). Data are the mean ± SD. Each dot represents one mouse in (b), (e), (g) and (h). one transcript (d) and (i), mean of duplicates from 3 mice in (f, right) and (k, right) (n = 3 animals examined over 3 independent experiments). Significance (P) was calculated with unpaired t-test or one-way ANOVA test in (g)-(h) and 2way ANOVA in (f)-(k). *: $P \leq 0.05$; ***: $P \leq 0.001$.

Supplementary Figure 6. Impaired mitochondrial metabolism increases basal glycolysis in GSH-deficient FoB.

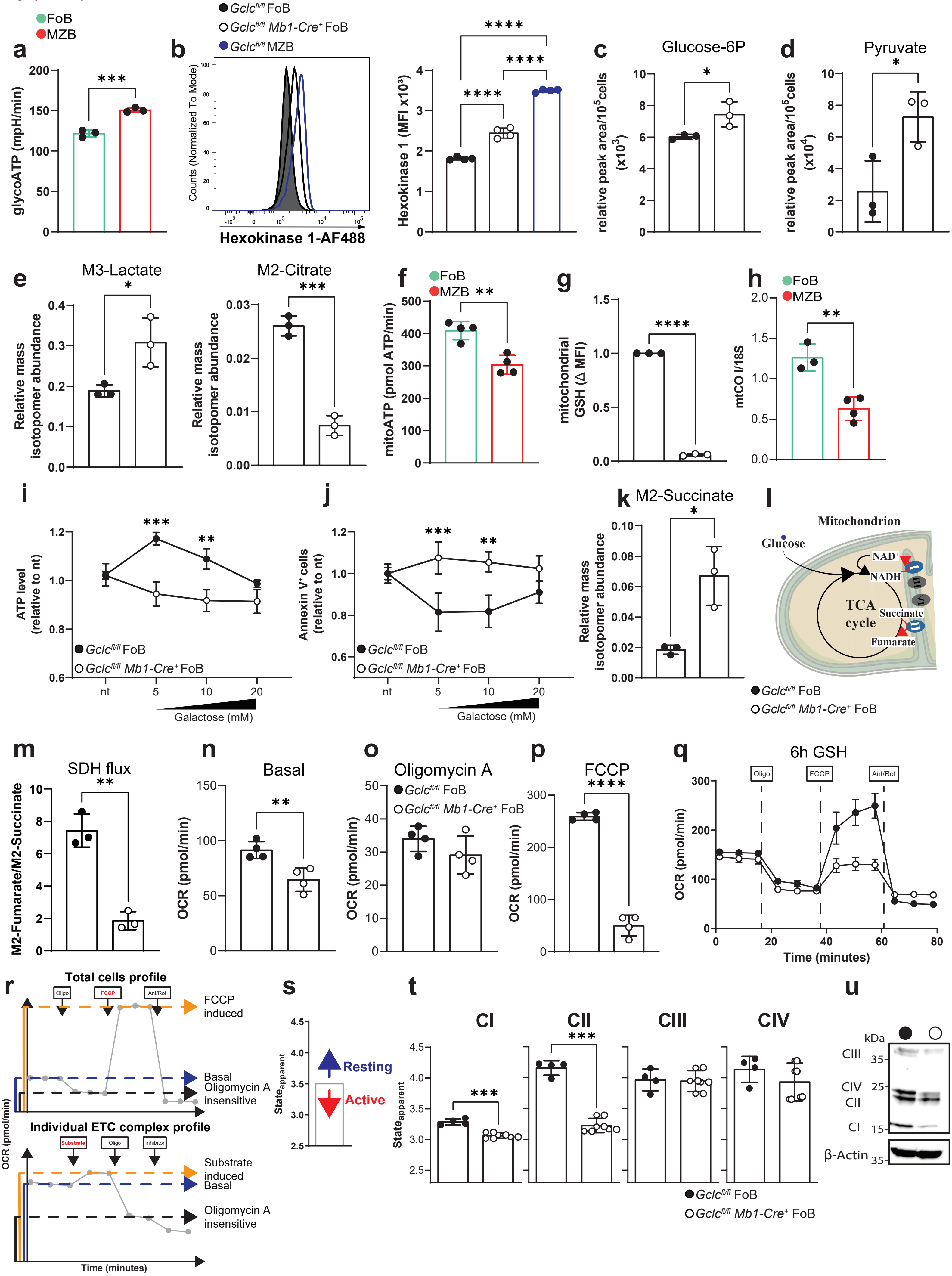

**Supplementary Figure 6. Impaired mitochondrial metabolism increases basal glycolysis in GSH-deficient FoB.**

**(a)** Seahorse quantitation of glycolysis-derived ATP in resting B6 FoB and MZB (n = 3 animals examined over 2 independent experiments). **(b)** Representative histogram (left) and quantitation (right) of immunostaining to detect Hexokinase-1 of *Gclc*$^{fl/fl}$ and *Gclc*$^{fl/fl}$ *Mb1-Cre*$^{+}$ FoB, including *Gclc*$^{fl/fl}$ MZB as control (gated as in Supplementary Figure 1a) (n = 4 animals examined over 3 independent experiments). **(c-d)** Quantitation of intracellular glucose-6P (c) and Pyruvate (d) level measured by LC-MS in resting *Gclc*$^{fl/fl}$ and *Gclc*$^{fl/fl}$ *Mb1-Cre*$^{+}$ FoB (n = 3 animals examined over 2 independent experiments). **(e)** Mass isotopomeric distribution (MID) of M3-lactate (left) and M2-Citrate (right) in *Gclc*$^{fl/fl}$ and *Gclc*$^{fl/fl}$ *Mb1-Cre*$^{+}$ FoB that were incubated with $^{13}$C-glucose and assayed at 5h post-activation with anti-IgM, CD40 ligand and IL-4 (n = 3 animals examined over 2 independent experiments). **(f)** Seahorse quantification of mitochondrial-derived ATP in resting B6 FoB and MZB (n = 3 animals examined over 2 independent experiments). **(g)** Flow-cytometry-based quantitation of monobromobimane (MBB) for the detection of GSH in purified Tom20$^{+}$ mitochondria from *Gclc*$^{fl/fl}$ and *Gclc*$^{fl/fl}$ *Mb1-Cre*$^{+}$ FoB (gated as in Supplementary Figure 1a) (n = 3 animals examined over 2 independent experiments). **(h)** Mitochondrial mass quantitation [ratio between mitochondrial cytochrome c oxidase subunit I (CO I) and nuclear 18S ribosomal RNA] as determined by RT-qPCR of DNA from B6 FoB and MZB (n = 3-4 animals examined over 3 independent experiments). **(i, j)** Normalized ATP levels (RLU) (i) and percentage of Annexin V-expressing cells (j), in *Gclc*$^{fl/fl}$ and *Gclc*$^{fl/fl}$ *Mb1-Cre*$^{+}$ FoB after 5h incubation with the indicated concentrations of galactose (n = 3 animals examined over 2 independent experiments). **(k)** MID of M2-succinate in *Gclc*$^{fl/fl}$ and *Gclc*$^{fl/fl}$ *Mb1-Cre*$^{+}$ FoB at 5h post-activation determined as in Fig. 5e (n = 3 animals examined over 2 independent experiments). **(l)** Ratio of M2-Fumarate/M2-succinate in *Gclc*$^{fl/fl}$ and *Gclc*$^{fl/fl}$ *Mb1-Cre*$^{+}$ FoB that were incubated with $^{13}$C-glucose and assayed at 5h post-activation (n = 3 animals examined over 2 independent experiments). **(m)** Complex I and II (CI and CII) reactions (red arrows) contribute to the enzymatic series of the TCA cycle within the mitochondria. **(n-p)** Quantitation of the OCR data in Fig. 6b at baseline (n) and upon treatment with oligomycin A (o) or FCCP (p) (n = 3-4 animals examined over 5 independent experiments). **(q)** Seahorse quantitation of OCR of *Gclc*$^{fl/fl}$ and *Gclc*$^{fl/fl}$ *Mb1-Cre*$^{+}$ FoB activated for 6h with anti-IgM, CD40 ligand and IL-4 and treated with 5mM GSH (n = 3-4 animals examined over 2 independent experiments).

Supplementary Figure 7. *In vivo* MZB-dependent antibody response and *in vitro* FoB activation properties are defective in *Gclc*-depleted mice.

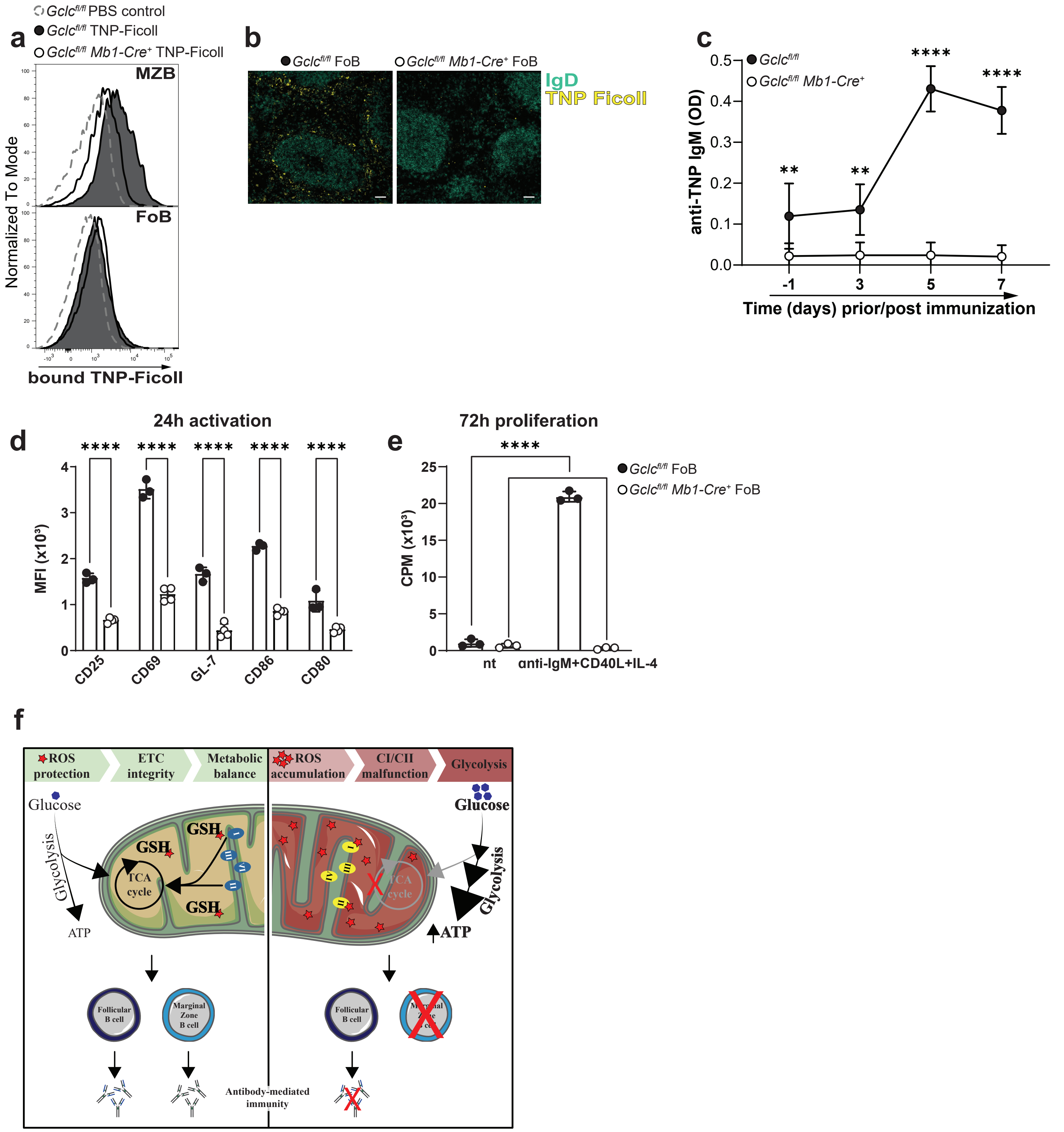

**Supplementary Figure 7. *In vivo* MZB-dependent antibody response and *in vitro* FoB activation properties are defective in *Gclc*-depleted mice.**

**(a)** Representative histogram of TNP capture by $Gclc^{fl/fl}$ and $Gclc^{fl/fl}$ *Mb1-Cre*$^{+}$ MZB and FoB (gated as in Fig. 2e) as measured by FACS (gated as in Supplementary Figure 1a) (n = 3 animals examined over 3 independent experiments). **(b)** Representative immunofluorescence staining to detect IgD (green) and TNP-Ficoll (yellow) in spleen follicles resected from one $Gclc^{fl/fl}$ and one $Gclc^{fl/fl}$ *Mb1-Cre*$^{+}$ mouse at 30min after *i.v.* injection of TNP-Ficoll. Scale bars, 50 µm (n = 3 animals examined over 3 independent experiments). **(c)** Titers of anti-TNP IgM as measured by ELISA in serum of $Gclc^{fl/fl}$ and $Gclc^{fl/fl}$ *Mb1-Cre*$^{+}$ mice at the indicated time points after *i.p.* injection of TNP-Ficol (n = 4 animals examined over 3 independent experiments). **(d-e)** Flow cytometry expression (MFI) of surface markers after 24h activation (d) (n = 3 animals examined over 2 independent experiments) and 3H-thymidine incorporation after 72h (e) of $Gclc^{fl/fl}$ and $Gclc^{fl/fl}$ *Mb1-Cre*$^{+}$ FoB with anti-IgM, CD40 ligand and IL-4 (n = 3 animals examined over 2 independent experiments). **(f)** Control of mitochondrial reactive oxygen species by the antioxidant glutathione (GSH) has a subset-specific role in murine B cells. GSH is identified as non-redundant antioxidant for the correct function of the electron transport chain, and it is required for antibody-mediated humoral immunity. Data are the mean ± SD. Each dot represents one mouse except for (c) (mean of duplicates from 4 mice). Data shown are representative of ≥3 independent experiments with 3-4 mice/group. Significance (P) was calculated with 2way ANOVA. **: $P \leq 0.01$; ***: $P \leq 0.0001$.
