# Supplementary material for: Glutathione-dependent redox balance characterizes the distinct metabolic properties of follicular and marginal zone B cells
Source: Nat Commun. 2022 Apr 4;13:1789. doi: 10.1038/s41467-022-29426-x (PMC8980022; doi:10.1038/s41467-022-29426-x)
Supplement: Supplementary file 2 — Description of additional Supplementary File [file 41467_2022_29426_MOESM2_ESM.pdf]

Descriptions of Additional Supplementary Data files

Supplementary Dataset 1 – This table contains a list of genes included in the GO: 0072593 relative to the MZB versus FoB comparison.

Supplementary Dataset 2 – This table contains the list of COMPASS reactions relative to the comparison MZB versus FoB (Gclc fl/fl).

Supplementary Dataset 3 - This table contains the list of COMPASS reactions relative to the comparison Gclc-deficient versus control FoB.

Supplementary Dataet 4 – This table contains the list of surface antibodies for flow cytometry used in the study.
