# Supplementary material for: Glutathione-dependent redox balance characterizes the distinct metabolic properties of follicular and marginal zone B cells
Source: Nat Commun. 2022 Apr 4;13:1789. doi: 10.1038/s41467-022-29426-x (PMC8980022; doi:10.1038/s41467-022-29426-x)
Supplement: Supplementary file 7 — Reporting Summary [file 41467_2022_29426_MOESM7_ESM.pdf]

nature portfolio

Corresponding author(s): Dirk Brenner

Last updated by author(s): 03/06/2022

Reporting Summary

Nature Portfolio wishes to improve the reproducibility of the work that we publish. This form provides structure for consistency and transparency in reporting. For further information on Nature Portfolio policies, see our Editorial Policies and the Editorial Policy Checklist.

Statistics

For all statistical analyses, confirm that the following items are present in the figure legend, table legend, main text, or Methods section.

n/a	Confirmed	
☐	☒	The exact sample size (*n*) for each experimental group/condition, given as a discrete number and unit of measurement
☐	☒	A statement on whether measurements were taken from distinct samples or whether the same sample was measured repeatedly
☐	☒	The statistical test(s) used AND whether they are one- or two-sided<br>*Only common tests should be described solely by name; describe more complex techniques in the Methods section.*
☒	☐	A description of all covariates tested
☒	☐	A description of any assumptions or corrections, such as tests of normality and adjustment for multiple comparisons
☐	☒	A full description of the statistical parameters including central tendency (e.g. means) or other basic estimates (e.g. regression coefficient) AND variation (e.g. standard deviation) or associated estimates of uncertainty (e.g. confidence intervals)
☐	☒	For null hypothesis testing, the test statistic (e.g. *F*, *t*, *r*) with confidence intervals, effect sizes, degrees of freedom and *P* value noted<br>*Give P values as exact values whenever suitable.*
☒	☐	For Bayesian analysis, information on the choice of priors and Markov chain Monte Carlo settings
☒	☐	For hierarchical and complex designs, identification of the appropriate level for tests and full reporting of outcomes
☐	☒	Estimates of effect sizes (e.g. Cohen's *d*, Pearson's *r*), indicating how they were calculated

Our web collection on statistics for biologists contains articles on many of the points above.

Software and code

Policy information about availability of computer code

Data collection	10x Genomics
Data analysis	R version 4.04, Seurat (v.4.0), Compass (Wagner et al. 2021 doi: https://doi.org/10.1016/j.cell.2021.05.045)

For manuscripts utilizing custom algorithms or software that are central to the research but not yet described in published literature, software must be made available to editors and reviewers. We strongly encourage code deposition in a community repository (e.g. GitHub). See the Nature Portfolio guidelines for submitting code & software for further information.

Data

Policy information about availability of data

All manuscripts must include a data availability statement. This statement should provide the following information, where applicable:

- Accession codes, unique identifiers, or web links for publicly available datasets
- A description of any restrictions on data availability
- For clinical datasets or third party data, please ensure that the statement adheres to our policy

The statement is included in the manuscript. The CITE-seq data from control and Gclc-deficient B cells have been deposited in GEO under the GSE194419; https://www.ncbi.nlm.nih.gov/geo/query/acc.cgi?acc=GSE194419.

Field-specific reporting

Please select the one below that is the best fit for your research. If you are not sure, read the appropriate sections before making your selection.

☒ Life sciences ☐ Behavioural & social sciences ☐ Ecological, evolutionary & environmental sciences

For a reference copy of the document with all sections, see nature.com/documents/nr-reporting-summary-flat.pdf

Life sciences study design

All studies must disclose on these points even when the disclosure is negative.

Sample size	Samples sizes were calculated based on power calculations .The Competence Center for Methodology and Statistics (CCMS) at the Luxembourg Institute of Health estimated an effect size ranging from 1 to 2.25, which will give a minimum power of 90% for the individual experiments.
Data exclusions	No data was excluded
Replication	2-5 (indicated in the figure legends)
Randomization	We used aged matched animals from littermates. Material and Methods: Animals. No randomization was used.
Blinding	Animal experiments were performed blinded.

Reporting for specific materials, systems and methods

We require information from authors about some types of materials, experimental systems and methods used in many studies. Here, indicate whether each material, system or method listed is relevant to your study. If you are not sure if a list item applies to your research, read the appropriate section before selecting a response.

Materials & experimental systems

n/a	Involved in the study	
☐	☒	Antibodies
☒	☐	Eukaryotic cell lines
☒	☐	Palaeontology and archaeology
☐	☒	Animals and other organisms
☒	☐	Human research participants
☒	☐	Clinical data
☒	☐	Dual use research of concern

Methods

n/a	Involved in the study	
☒	☐	ChIP-seq
☐	☒	Flow cytometry
☒	☐	MRI-based neuroimaging

Antibodies

Antibodies used

Antigen Fluorochrome Supplier (cat. number) Clone

CD11b Pacific Blue BioLegend (101224) M1/70
CD19 FITC BD (557398) 1D3
CD19 BV785 BioLegend (115543) 6D5
CD1d Pacific Blue BioLegend (123517) 1B1
CD21/CD35 FITC BioLegend (123408) 7,00E+09
CD21/CD35 APC BioLegend (123411) 7,00E+09
CD23 PE BioLegend (101608) B3B4
CD23 Pacific Blue BioLegend (101615) B3B4
CD24 APC Fisher Scientific (17-0242-82) M1/69
CD35 BUV737 BD (741751) 8C12
CD45R/B220 PE Immunostep (MO45RPE(V100)) RA3-6B2
CD93 BUV395 BD (740275) AA4.1
CD95 BV421 BD (562633) Jo2
GL-7 AF647 BioLegend (144606) GL7
IgD BV605 BD (63003) 11-26C.1
IgM PE/Cy7 BioLegend (406514) RMM-1
TCR beta APC Fisher Scientific (15380800) H57-597
IgM BUV395 BD (566217) R6-60.2
CD93 BUV737 BD (741800) AA4.1

	ICAM-1 APC Miltenyi Biotec (130-104-248) REA171<br>VCAM-1 PE Fisher Scientific (15586846) 429
Validation	Antibodies were validated using FMO controls. In addition the selection of antibodies was based on clones that are well known in the field.

Animals and other organisms

Policy information about studies involving animals; ARRIVE guidelines recommended for reporting animal research

Laboratory animals	species: mus musculus, strains: C57BL/6, Gclc fl/fl (Chen et al. 2007 doi.org/10.1002/hep.21635), B6.C(Cg)-Cd79atm1(cre)Reth/EhobJ (Mb1-Cre). This study involved male and female mice at an age of 7-12 weeks. Age- and sex-matched mice were used for all experiments. The mice were housed and bred under specific pathogen-free (SPF) conditions at the Luxembourg Institute of Health (LIH) and the BTA facility of the University of Luxembourg.
Wild animals	The study did not involve wild animals.
Field-collected samples	The study did not involve samples collected from the field.
Ethics oversight	Luxembourg Institute of Health’s Animal Welfare Structure (AWS).

Note that full information on the approval of the study protocol must also be provided in the manuscript.

Flow Cytometry

Plots

Confirm that:

- [x] The axis labels state the marker and fluorochrome used (e.g. CD4-FITC).
- [x] The axis scales are clearly visible. Include numbers along axes only for bottom left plot of group (a 'group' is an analysis of identical markers).
- [x] All plots are contour plots with outliers or pseudocolor plots.
- [x] A numerical value for number of cells or percentage (with statistics) is provided.

Methodology

Sample preparation	For surface staining cells were incubated at 4°-8°C, in the dark with fluorochrome-conjugated CD19, CD23, CD21-CD35 or CD35, IgD, IgM, CD1d, CD24, CD93, CD95, GL-7. The antibody mix was washed from the cells after 20-30min. Samples from in vivo LCMV experiments were fixed for 10min at RT in a volume of at least 100µL 2% formaldehyde (FA) after staining.
Instrument	BD Fortessa, Novocyte Quanteon
Software	FlowJo 10.6.1
Cell population abundance	FoB: 70-80%<br>MZB: 5-10%<br>Purity > 90%
Gating strategy	FSC, SSC, doublet exclusion + live cell staining

- [x] Tick this box to confirm that a figure exemplifying the gating strategy is provided in the Supplementary Information.
